# Genome-Wide Expression Profiling of *OsWRKY* Superfamily Genes during Infection with *Xanthomonas oryzae* pv. *oryzae* Using Real-Time PCR

**DOI:** 10.3389/fpls.2017.01628

**Published:** 2017-09-20

**Authors:** Nae Young Choi, Eunhye Lee, Sang Gu Lee, Chang Hyun Choi, Sang Ryeol Park, Ilpyung Ahn, Shin Chul Bae, Cheol Ho Hwang, Duk-Ju Hwang

**Affiliations:** ^1^National Institute of Agricultural Science, Rural Development Administration Jeonju, South Korea; ^2^Department of Crop Science and Biotechnology, Dankook University Cheonan, South Korea

**Keywords:** OsWRKY transcription factors superfamily, expression profiling, *Xanthomonas oryzae* pv. *oryzae*

## Abstract

WRKY transcription factors (TFs) are involved in regulating a range of biological processes such as growth, development, and the responses to biotic and abiotic stresses. Genome-wide expression profiling of *OsWRKY* TF superfamily genes in rice after infection with *Xanthomonas oryzae* pv. *oryzae* (*Xoo*) was performed to elucidate the function of OsWRKY TFs in the interaction between rice and *Xoo*. Of the 111 *OsWRKY* TF genes tested, the transcription of 94 genes changed after *Xoo* infection. The *OsWRKY* TF genes were classified into eight types according to their expression profiles. Eighty-two genes in Groups I, II, III, IV, VII were up-regulated after exposure to a compatible or an incompatible race of *Xoo*. Examination of salicylic acid (SA)-deficient rice lines revealed that SA was involved in Xa1-mediated resistance to *Xoo* infection. *OsWRKY* TF genes involved in Xa1-mediated resistance were classified according to their SA-dependent or -independent expression. In SA-deficient rice, the expression of 12 of 57 OsWRKY TF genes involved in Xa1-mediated resistance was compromised. Of these six OsWRKY TF genes were induced by SA. *OsWRKY88*, an example of a gene possibly involved in SA-dependent Xa1-mediated resistance, activated defense related genes and increased resistance to *Xoo*. Thus, expression profiling of *OsWRKY* TF genes may help predict the functions of *OsWRKY* TF genes involved in Xa1-mediated resistance.

## Introduction

WRKY transcription factors (TFs) are characterized by the presence of one or two 60-amino-acid WRKY motifs, a very highly conserved WRKYGQK sequence, and zinc finger-like motifs [Cys(2)-His(2) or Cys(2)-HisCys] that bind to the W-box [TTGAC(C/T)] cis-element in target gene promoters (Sun et al., 2003; Cai et al., 2008; Ciolkowski et al., 2008; Pandey and Somssich, 2009). WRKY TFs are classified into three groups (Groups I–III) according to the similarity of their WRKY motifs (Zhang and Wang, 2005). WRKY TF families in *Arabidopsis* and rice contain 72 and 125 members, respectively (Eulgem et al., 2000; Rice WRKY Working Group., 2012).

Numerous WRKY TFs are involved in responding to biotic stresses in rice and *Arabidopsis* (Dong et al., 2003; Ryu et al., 2006; Berri et al., 2009). Several studies described expression profiling of *OsWRKY* TF genes upon infection with pathogens such as *Magnaporthe oryzae* and *Xanthomonas oryzae* pv. *oryzae* (*Xoo*) in rice (Ryu et al., 2006; Bagnaresi et al., 2012; Wei et al., 2013). The first such study examined the expression of 45 *OsWRKY* TF genes in response to *M. oryzae* challenge and found that 15 *OsWRKYs* were up-regulated upon infection (Ryu et al., 2006). Later research with a custom OsWRKYARRAY found that 18 of 104 *OsWRKY* TF genes were up-regulated upon *M. oryzae* infection (Berri et al., 2009). A recent transcriptome study in rice noted that *OsWRKY* TF genes were significantly enriched in the group of genes up-regulated during the early defense response to *M. oryzae* infection and that OsWRKY47 played a critical role in rice blast resistance (Wei et al., 2013).

To date, relatively few studies have examined *OsWRKY* TF superfamily gene expression upon infection with *Xoo*, but several studies have examined individual *OsWRKY* TF genes in *Xoo-*mediated resistance. One study reported that 9 of 45 *OsWRKY* genes, namely, *OsWRKY7, 10, 11, 30, 32, 67, 70, 83* [renamed *94* by the Committee on Gene Symbolization, Nomenclature, and Linkage (CGSNL)], and *85* (renamed *96* by CGSNL) were up-regulated upon infection with *Xoo* (Ryu et al., 2006). The expression profiles of some *OsWRKY* genes were summarized by Jimmy and Babu (Jimmy and Babu, 2015). Other studies observed up-regulation of *OsWRKY6, 12, 13, 30, 45-1, 71*, and *76* in response to *Xoo* infection (Liu et al., 2005, 2007; Chujo et al., 2008; Qiu and Yu, 2009; Tao et al., 2009; Hwang et al., 2011; Lee et al., 2013; Choi et al., 2015). However, no comprehensive genome-wide expression data are available for all 125 known *OsWRKY* superfamily genes.

SA has been shown to play a key role in plant immunity (Delaney et al., 1994; Jimmy and Babu, 2015). Many WRKY genes from a number of plants have also been shown to be induced by SA (Dong et al., 2003; Ryu et al., 2006). Transgenic plants expressing the bacterial *NahG* gene encoding a salicylate hydroxylase that converts SA to catechol are often used to evaluate the role of SA in plant immunity (Delaney et al., 1994; Yang et al., 2004). Pathogen mediated WRKY gene expression has also been examined in *NahG Arabidopsis* plants (Dong et al., 2003). To date there is no report on SA effect using *NahG* transgenic rice plants in pathogen mediated *OsWRKY* gene expression yet.

Nine resistance (R) genes for *Xoo*, namely, *Xa1, Xa3/26, xa5, Xa10, xa13, Xa21, Xa23, xa25*, and *Xa27*, were cloned previously (Song et al., 1995; Yoshimura et al., 1998; Iyer and McCouch, 2004; Sun et al., 2004; Gu et al., 2005; Chu et al., 2006; Liu et al., 2011; Tian et al., 2014; Wang C. et al., 2015). Most proteins encoded by plant disease R genes share common structural features such as nucleotide-binding site leucine-rich repeat (NBS-LRR) regions. Of the nine *Xoo* R genes cloned to date, only *Xa1* encodes a typical NBS-LRR-type protein. The mechanisms underpinning Xa1-mediated resistance remain largely unknown. In this study, genome-wide expression profiling of *OsWRKY* superfamily genes was used to investigate Xa1-mediated defense mechanisms and the function of *OsWRKY88*, an example of a gene possibly involved in SA-dependent Xa1-mediated resistance, was analyzed.

## Materials and methods

### Plant material and pathogen treatment

Rice seedlings (*Oryza sativa* L. Japonica cv. Ilmi) carrying the Xa1 R gene were grown in a greenhouse at 28°C for 3 weeks. Seedlings were inoculated with compatible (KACC10859) or incompatible (KXO42) *Xoo* strains grown on PSA medium (10 g/L peptone, 10 g/L sucrose, 1 g/L sodium glutamate, and 15 g/L agar) for 2 days until OD_600_ = 0.5 (Kim et al., 2013). Leaves were harvested at 0, 6, 12, and 24 hpi. Seedlings were treated with 1 mM SA, and leaves were harvested at 0, 12, 24 h after treatment (hat). All samples were frozen in liquid nitrogen immediately after harvesting and stored at −80°C until used.

### Quantitative RT-PCR

The 125 *OsWRKY* TF superfamily genes were reported by CGSNL (Rice WRKY Working Group., 2012; Table S1). Among them we could not obtain the data for the 14 OsWRKY TFs in this study due to various reasons. Primers for qRT-PCR were designed using the rice genome annotation database (http://rice.plantbiology.msu.edu; Tables S1, S2). Nucleotide sequences for OsWRKY44 and OsWRKY59 were not found in the database. Some OsWRKY TFs are duplicated genes: *OsWRKY46 (91), 61 (103), 4 (122)*. NCBI primer-blast (https://www.ncbi.nlm.nih.gov/tools/primer-blast/) was used to optimize the design of specific primers for each *OsWRKY* TF gene. For nine OsWRKY TFs (*OsWRKY24, 33, 35, 57, 61, 92, 99, 116, 124*) we could not optimize the condition to get PCR products. Specific qRT-PCR primers for *OsWRKY* TF genes are listed in Table S1.

Total RNA was extracted from harvested leaves using Trizol reagent (Invitrogen, USA). M-MLV reverse transcriptase (Promega, USA) was used to synthesize cDNA from total RNA (2 μg). qRT-PCR was conducted as described by Kim et al. (2013). OsActin was used as a calibration control to determine OsWRKY gene expression levels (Schmittgen and Livak, 2008). The 2^−ΔΔct^ method was used for analysis of relative gene expression. Three biological replicates were performed for each qRT-PCR analysis. Above 2-fold difference at each time point in each expression profile was considered as either increase or decrease of OsWRKY expression.

### Generation of transgenic rice plants

Gateway cloning using pB2GW7 (Karimi et al., 2002) was used to generate constructs 35S::NahG from *Arabidopsis NahG* plants and 35S::OsWRKY88. *Agrobacterium tumefaciens* LBA4404 was transformed with the 35S::NahG construct and the 35S::OsWRKY88 construct. *A. tumefaciens* carrying both constructs was used to transform rice embryogenic calli (Kim et al., 2009). Transgenic rice T_0_ plants were selectively screened using 0.1% Bastar™ spray. Integration of the construct was confirmed by PCR of genomic DNA with bar-specific primer sets (Table S2). Homozygous *NahG* transgenic rice plants were confirmed by 0.1% Bastar™ spray through several generations and used for disease assays and qRT-PCRs. In the case of OsWRKY88 ox lines we used T2 lines selected by 0.1% Bastar™ spray.

### Plant disease assays

*Xoo* KXO42 and KACC10859 bacteria were grown overnight at 28°C in PSA medium and resuspended at 10^8^ cells/mL in 10 mM MgCl_2_. The scissors-dip method (Kauffman et al., 1973) was used to challenge WT and ten SA-deficient rice plants with *Xoo* (KXO42) and WT and ten OsWRKY88 ox lines (T_2_) with *Xoo* (KACC10859). Disease was assessed as the average lesion length in leaves of individual plants 14 days post-inoculation (dpi). Bacterial growth was assessed at 0, 7, and 14 dpi (Shimono et al., 2007).

### Promoter transient expression assays

A 2 kb region of the *CHITINASE 2* (*CHIT2*; Os04g41620) promoter was amplified by PCR using promoter-specific primers (Table S2) and introduced into an entry vector, pENTR/d-TOPO (Invitrogen, Carlsbad, CA, USA). The *pCHIT2::GFP-GUS* construct was made an LR reaction between the entry clones containing the promoter and the destination vector pBGWFS7 (Karimi et al., 2002). Transient expression assays in *Nicotiana benthamiana* were performed using the protocol reported previously (Li, 2011). *N. benthamiana* was infiltrated with an *Agrobacterium* carrying *pCHIT2::GFP-GUS* and *pPR10a::GFP-GUS* (Hwang et al., 2008) constructs alone, or with a mixture of *Agrobacterium* carrying *35S::OsWRKY88* and either *pCHIT2::GFP-GUS* or *pPR10a::GFP-GUS*. Infiltrated leaves were collected 2 days post-infiltration, and promoter activities in each sample were visualized using β-glucuronidase (GUS) activity assay and staining.

## Results

### Expression profiles of OsWRKY TF superfamily genes during *Xoo* infection were analyzed

**Figure 1:**
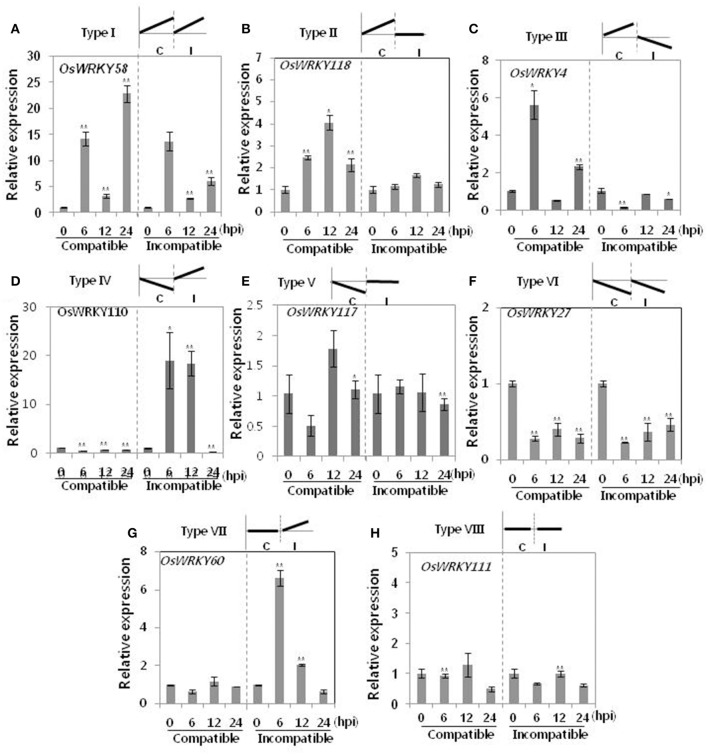
Expression profiling of *OsWRKY* TF genes upon infection with compatible and incompatible races of *Xoo*. **(A)** Type I: Genes up-regulated after challenge with both compatible and incompatible *Xoo* races. **(B)** Type II: Genes up-regulated only after challenge with a compatible *Xoo* race. **(C)** Type III: Genes up-regulated after challenge with a compatible *Xoo* race and down-regulated by infection of incompatible *Xoo* race. **(D)** Type IV: Genes down-regulated after challenge with a compatible *Xoo* race and up-regulated by infection of incompatible *Xoo* race. **(E)** Type V: Genes down-regulated only after challenge with an incompatible *Xoo* race. **(F)** Type VI: Genes up-regulated after challenge with both compatible and incompatible *Xoo* races. **(G)** Type VII: Genes up-regulated only after challenge with an incompatible *Xoo* race. **(H)** Type VIII: Genes exhibiting no expression response upon challenge with either a compatible or an incompatible *Xoo* race. Asterisks indicate significant differences (^**^P < 0.01; ^*^P < 0.05).

The roles of *OsWRKY* TF superfamily genes in the defense pathway mediated by Xa1, a bacterial blight resistance protein (Yoshimura et al., 1998), were investigated using expression profiling. *Oryza sativa* L spp. *japonica* cv. Ilmi, which carried the *Xa1* gene, was profiled after challenge with an incompatible (KXO42) or a compatible (KACC10859) race of *Xoo*. *OsWRKY* TF superfamily genes used in this study were named according to recently re-defined nomenclature (Rice WRKY Working Group., 2012; Table S1). Expression profiles of *OsWRKY* TF superfamily genes were shown in eight different types and the results were summarized in Table 1 (Types I–VIII). The expression profile of a representative OsWRKY TF in each type was shown in Figure 1 and the rest of them were shown in Figure S1. Most of the *OsWRKY* TFs (57 genes) were up-regulated in response to both the compatible and incompatible *Xoo* races (Type I). Of these, *OsWRKY58* transcription was particularly elevated upon challenge with *Xoo* (Figure 1A). *OsWRKY* TFs (*OsWRKY107, 118*) showed Type II pattern that were up-regulated only in response to the compatible *Xoo* race (Table 1). Of these, transcription of *OsWRKY118* was noticeably elevated 6 hpi (Figure 1B). Fourteen *OsWRKY* TFs (*OsWRKY4, 5, 14, 19, 28, 48, 78, 79, 82, 85, 86, 98, 101, 121*) showed Type III pattern that up-regulated in response to the compatible *Xoo* race and down-regulated in response to the incompatible *Xoo* race. Of these transcription of *OsWRKY4* was noticeably elevated 6 hpi (Figure 1C). Six OsWRKY TFs (OsWRKY74, 77, 110, 119, 120) showed Type IV pattern that were down-regulated in response to the compatible *Xoo* race and up-regulated in response to the incompatible *Xoo* race. Of these OsWRKY110 was decreased at 3 hpi upon the infection of the compatible *Xoo* race and increased at 12 hpi upon the infection of the incompatible *Xoo* race (Figure 1D). Two OsWRKY TFs showed Type V pattern that were down regulated only upon infection of the compatible *Xoo* race. Of these OsWRKY117 was decreased at 6 hpi (Figure 1E). Twenty two OsWRKY TFs showed Type VI pattern that was down regulated both upon infection of the compatible and incompatible *Xoo* race. Of these, *OsWRKY27* was dramatically down-regulated 6 hpi with both the compatible and incompatible interactions (Figure 1F). Type VII contained three *OsWRKY* TFs (OsWRKY32, 46, 60) that were up-regulated only in response to only the incompatible *Xoo* race. Of these, the *OsWRKY60* transcript reached maximum levels 6 h post-inoculation (hpi; Figure 1G). Finally, Type VIII contained five *OsWRKY* TFs (OsWRKY53, 72, 102, 111, 125) whose transcription profiles were unaffected by *Xoo* infection (Figure 1H). For the reasons described in the Materials and Methods section of qRT-PCR and Table 1, no expression profile data were obtained for 14 *OsWRKY* genes (OsWRKY24, 33, 35, 44, 57, 59, 61, 91, 92, 99, 103, 116, 122, 124).

**Table 1:** Expression profiles of *OsWRKY* TF genes in response to *Xoo* infection were summarized.

**Types**	**Expression profile**	**OsWRKY TFs**
I	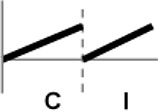	*OsWRKY1, 2, 3, 6, 7, 8, 9, 10, 11, 12, 13, 16, 18, 22, 23, 25, 26, 29, 30, 36, 38, 40, 42, 43, 45, 47, 49, 50, 51, 52, 54, 55, 58, 63, 64, 65, 66, 69, 70, 71, 76, 80, 81, 84, 87, 88, 90, 94, 96, 105, 106, 108, 112, 113, 114, 115, 123*
II	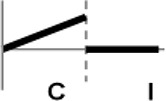	*OsWRKY107, 118*
III	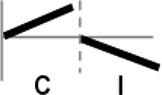	*OsWRKY4, 5, 14, 19, 28, 48, 78, 79, 82, 85, 86, 98, 101, 121*
IV	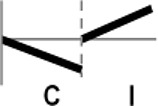	*OsWRKY56, 74, 77, 110, 119, 120*
V	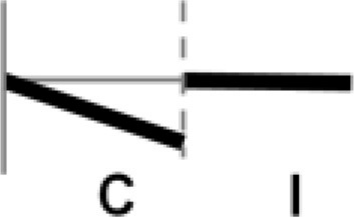	*OsWRKY72, 117*
VI	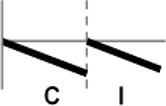	*OsWRKY15, 17, 20, 21, 27, 31, 34, 37, 39, 41, 54, 56, 62, 67, 68, 73, 75, 83, 93, 95, 104, 109*
VII	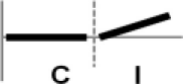	*OsWRKY32, 46, 60*
VIII	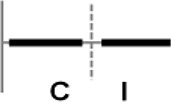	*OsWRKY53, 72, 111, 102, 125*

*No data were generated for the following OsWRKY TF genes*.

*1. Nucleotide sequences for OsWRKY44 and OsWRKY59 were not found*.

*2. Duplicated genes: OsWRKY91 (46), 103 (61), 122 (4)*.

*3. No PCR products were obtained: OsWRKY24, 33, 35, 57, 61, 92, 99, 116, 124*.

### Salicylic acid plays a role in Xa1-mediated resistance

**Figure 2:**
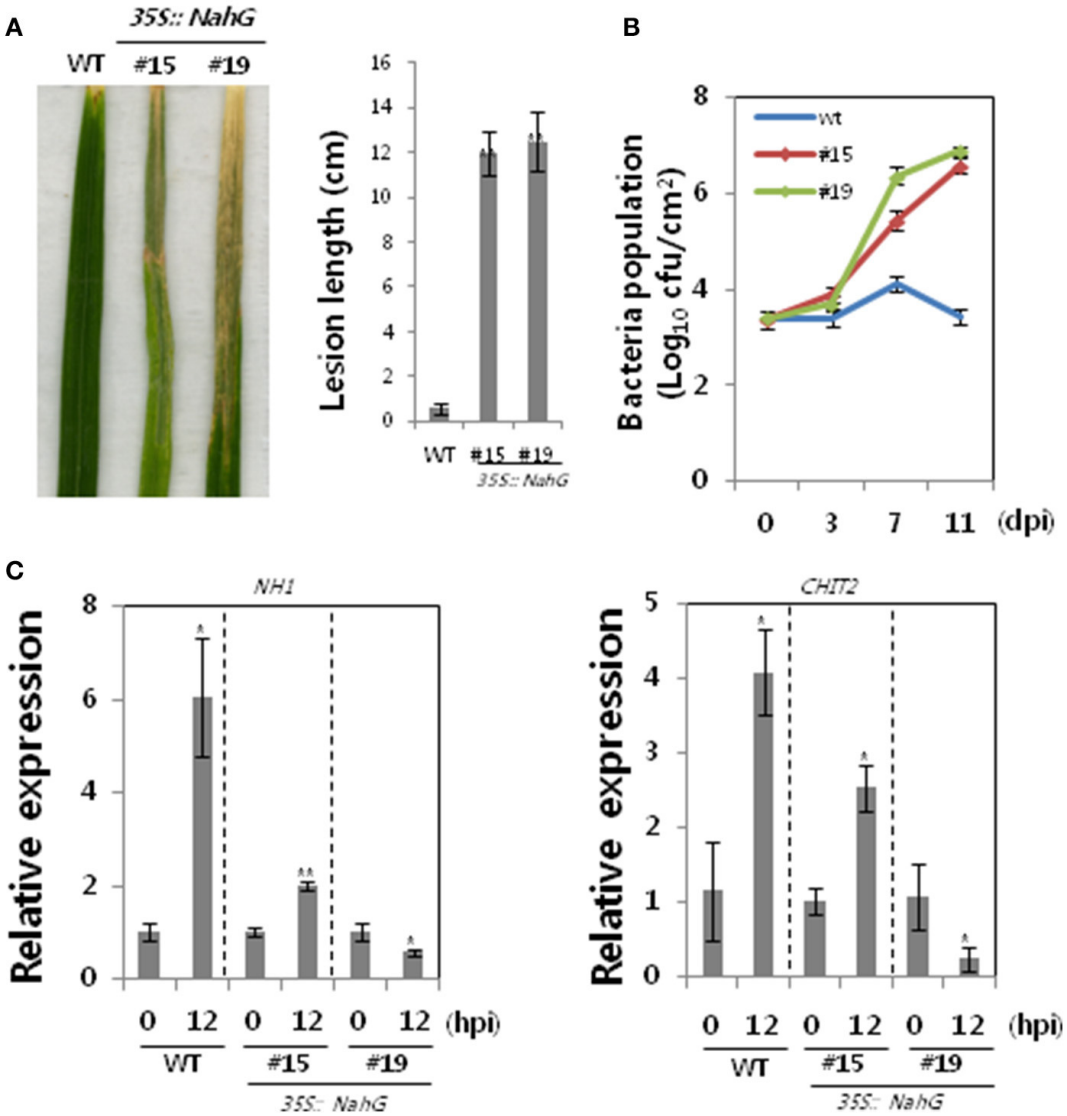
Disease assays of SA-deficient rice plants infected with an incompatible *Xoo* race. **(A)** WT (Ilmi cultivar carrying Xa1) and SA-deficient rice plants derived from the Ilmi cultivar were challenged with an incompatible race of *Xoo*. Lesion lengths were measured at 14 dpi. **(B)** Bacterial growth was assessed at 3 and 11 dpi. **(C)** qRT-PCR expression analysis of genes encoding NH1 and chitinase in WT and SA-deficient rice lines 12 h after challenge with an incompatible race of *Xoo*. Asterisks indicate significant differences (^**^P < 0.01; ^*^P < 0.05).

Transcription of 66 *OsWRKY* TF genes (Groups I, IV, and VII) was up-regulated during the incompatible interaction of rice cultivar Ilmi carrying Xa1 with *Xoo*, suggesting that many OsWRKY TFs could be involved in the Xa1-mediated defense pathway. Salicylic acid (SA) has a role in plant defense response, and SA-deficient transgenic rice plants were therefore used to investigate the involvement of SA in Xa1-mediated defense. Bacterial salicylate hydroxylase, which is encoded by the *NahG* gene, degrades SA to catechol. SA-deficient transgenic rice plants were generated by introduction of the *NahG* gene into rice via *Agrobacterium*-mediated transformation. Nineteen independent *NahG*-expressing transgenic lines were produced. Lines #15 and #19 were selected for further analysis based on their *NahG* transcript levels (data not shown). Transgenic lines challenged with incompatible *Xoo* race KXO42 exhibited longer lesions (~13 cm) than wild-type (WT) Ilmi plants (~0.2 cm; Figures 2A,B), indicating that Xa1-mediated resistance was compromised in the transgenic plants. Bacterial populations in the two SA-deficient lines were approximately higher than in the WT at 3, 7, and 11 dpi, respectively (Figure 2B). Activation of the Xa1-mediated defense pathway in the SA-deficient lines was examined using qRT-PCR transcriptional analysis of the genes encoding *NH1/OsNPR1* and *chitinase 2* (*CHIT2*). Compared with the WT, induction of both genes was partially compromised in line #15 and severely compromised in line #19 (Figure 2C). These observations correlated with lesion length and bacterial growth data. On the other hand, some studies claim that catechol results in susceptibility to *Magnaporthe grisea* but not to *Striga hermonthica* (Yang et al., 2004; Mutuku et al., 2015). We therefore examined the effect of catechol on *Xoo* infection (Figure S1), which revealed it had no effect on compatible or incompatible *Xoo*-rice interactions. Thus, impaired defense in SA-deficient lines is not due to catechol production. Overall, these results suggest that SA plays a role in Xa1-mediated resistance.

### SA-dependent transcriptional induction of OsWRKY TFs up-regulated in the Xa1-mediated defense pathway

**Figure 3:**
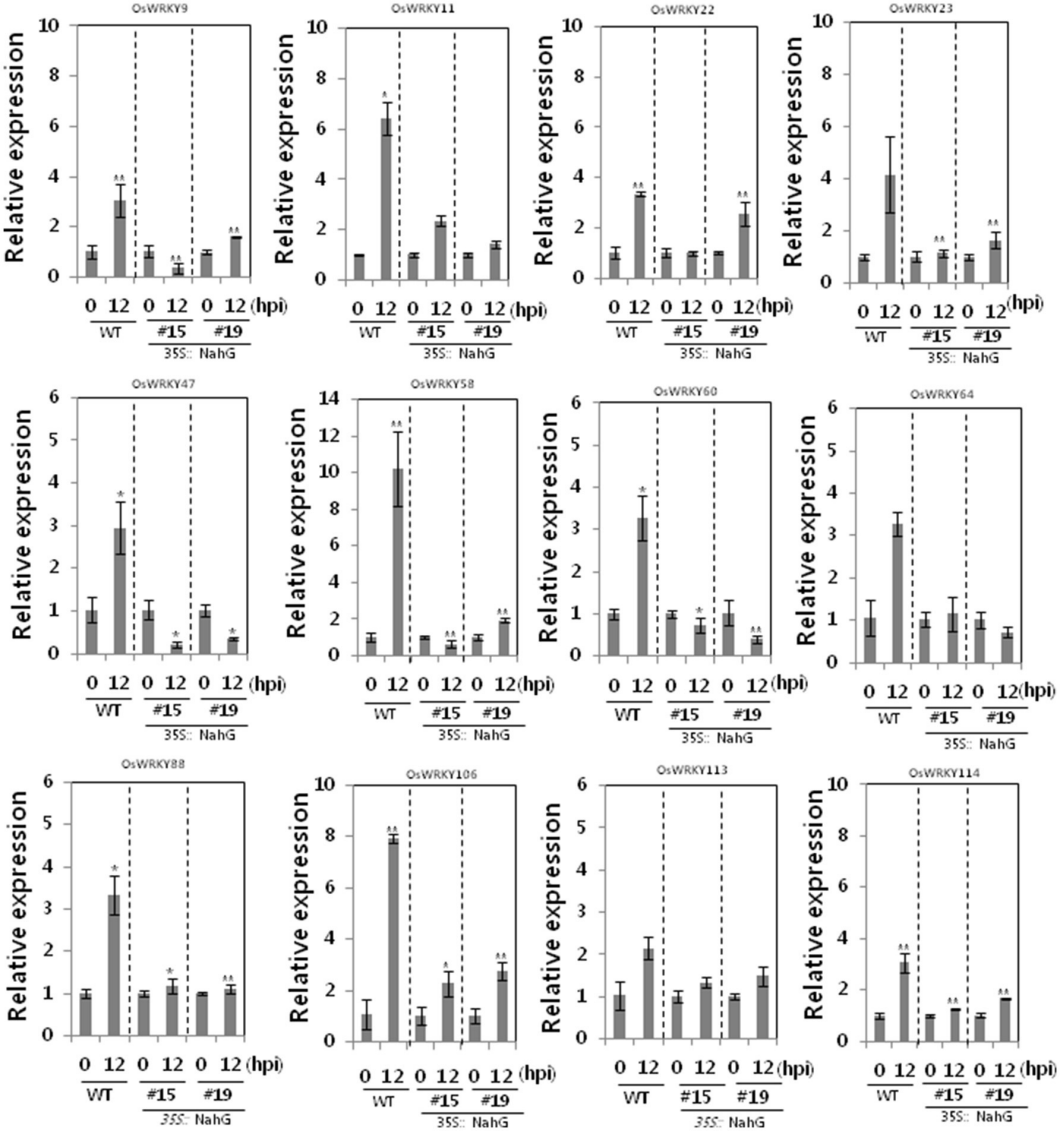
Expression of OsWRKY TF genes up-regulated by infection of an incompatible *Xoo* race in SA-deficient rice plants. qRT-PCR expression analysis of *OsWRKY* genes in WT and SA-deficient rice lines 12 h after challenge with an incompatible race of *Xoo*. Asterisks indicate significant differences (^**^P < 0.01; ^*^P < 0.05).

66 *OsWRKY* TF genes (Groups I, IV, and VII) were up-regulated after challenge with the incompatible *Xoo* race (Table 1). Type I expression pattern is further divided into Type Ia and Ib. *OsWRKY* TF genes in Type Ia exhibited more pronounced up-regulation (either a faster response or a more abundant response) with the incompatible interaction than with the compatible interaction, whereas *OsWRKY* TF genes in Type Ib exhibited a more pronounced effect with the compatible interaction than with the incompatible interaction. *OsWRKY* TF genes in Type Ia (Table 2) exhibited expression profiles typical of defense response genes, and this group was therefore used to further investigate SA-dependent Xa1-mediated resistance. Expression of *OsWRKY* TF genes in Type Ia was investigated in SA-deficient rice plants using qRT-PCR (Figure 3). Gene expression during the incompatible interaction was not compromised in the SA-deficient rice lines for the majority of Type Ia *OsWRKY* TF genes (34 genes). However, induction of *OsWRKY9, 11, 22, 23, 47, 58, 60, 64, 88, 106, 113*, and *114* expression during the incompatible interaction was reduced in the two SA-deficient rice lines. This suggests that *OsWRKY9, 11, 22, 23, 47, 58, 60, 64, 88, 106, 113*, and *114* were induced in a SA-dependent manner as part of the Xa1-mediated defense pathway but that other *OsWRKY* TF genes in Type 1a were induced as part of the Xa1 mediated response in a SA-independent manner.

**Table 2:** Expression of *OsWRKY* TF genes in SA-deficient rice after challenge with an incompatible race of *Xoo* assessed by qRT-PCR.

**Type I**	**Expression pattern**	**Gene**
OsWRKY2, 6, 7, 8, 9, 11, 12, 18, 22, 23,25 ,26, 29, 42, 45, 47, 50, 52, 55, 58, 60, 64, 65, 66,76, 80, 84, 88, 105, 106, 108, 112, 113, 114	Compromised in SA-deficient rice compared with the WT	OsWRKY9, 11, 22, 23, 47, 58, 60, 64, 88, 106, 113, 114
	Unchanged in SA-deficient rice compared with the WT	OsWRKY2, 7, 12, 18, 25, 26, 42, 45, 50, 52, 55, 65, 66, 76, 80, 84, 105, 108, 112

*Type I OsWRKY TF genes were further classified into Type Ia and Ib. Type Ia contained OsWRKY TF genes that exhibited more pronounced up-regulation with the incompatible interaction than with the compatible interaction. Type Ib contained OsWRKY TF genes that exhibited more pronounced up-regulation with the compatible interaction than with the incompatible interaction*.

**Figure 4:**
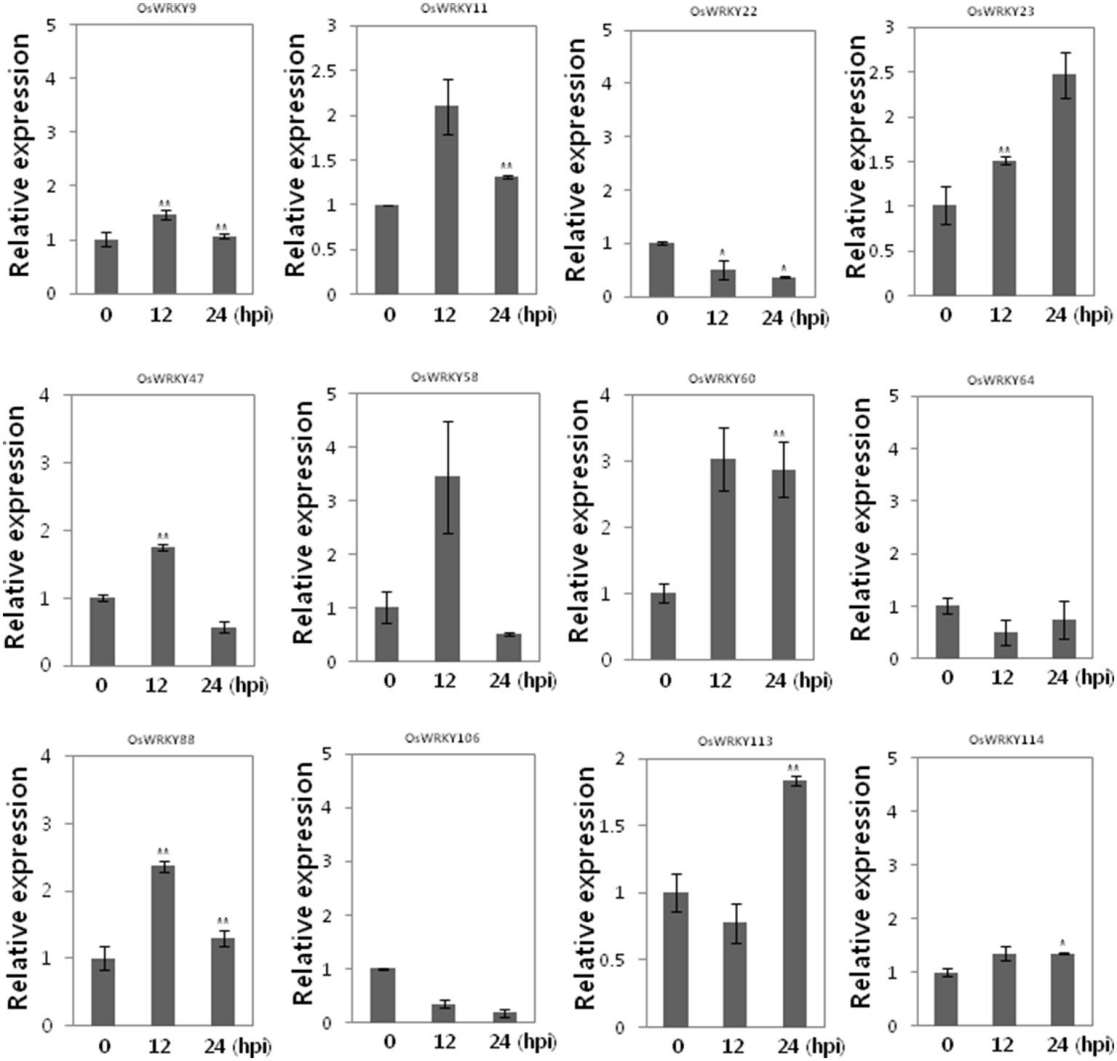
Expression of 12 *OsWRKY* TF genes in response to SA. Expression analysis of 12 *OsWRKY* genes in WT at 0, 12, 24 h after SA treatment. Asterisks indicate significant differences (^**^P < 0.01; ^*^P < 0.05).

To investigate whether these *OsWRKY* TFs are induced by SA we performed qRT-PCR (Figure 4). Among 12 *OsWRKY* TFs *OsWRKY11, 23, 47, 58, 60, 88* were induced by SA. However, *OsWRKY9, 22, 64, 106, 113, 114* were not induced by SA. Taken together the six *OsWRKY* TFs appears to play roles in Xa1 mediated resistance.

### Ectopic expression of *OsWRKY88*, a type I OsWRKY TF that enhanced resistance to *Xoo*

**Figure 5:**
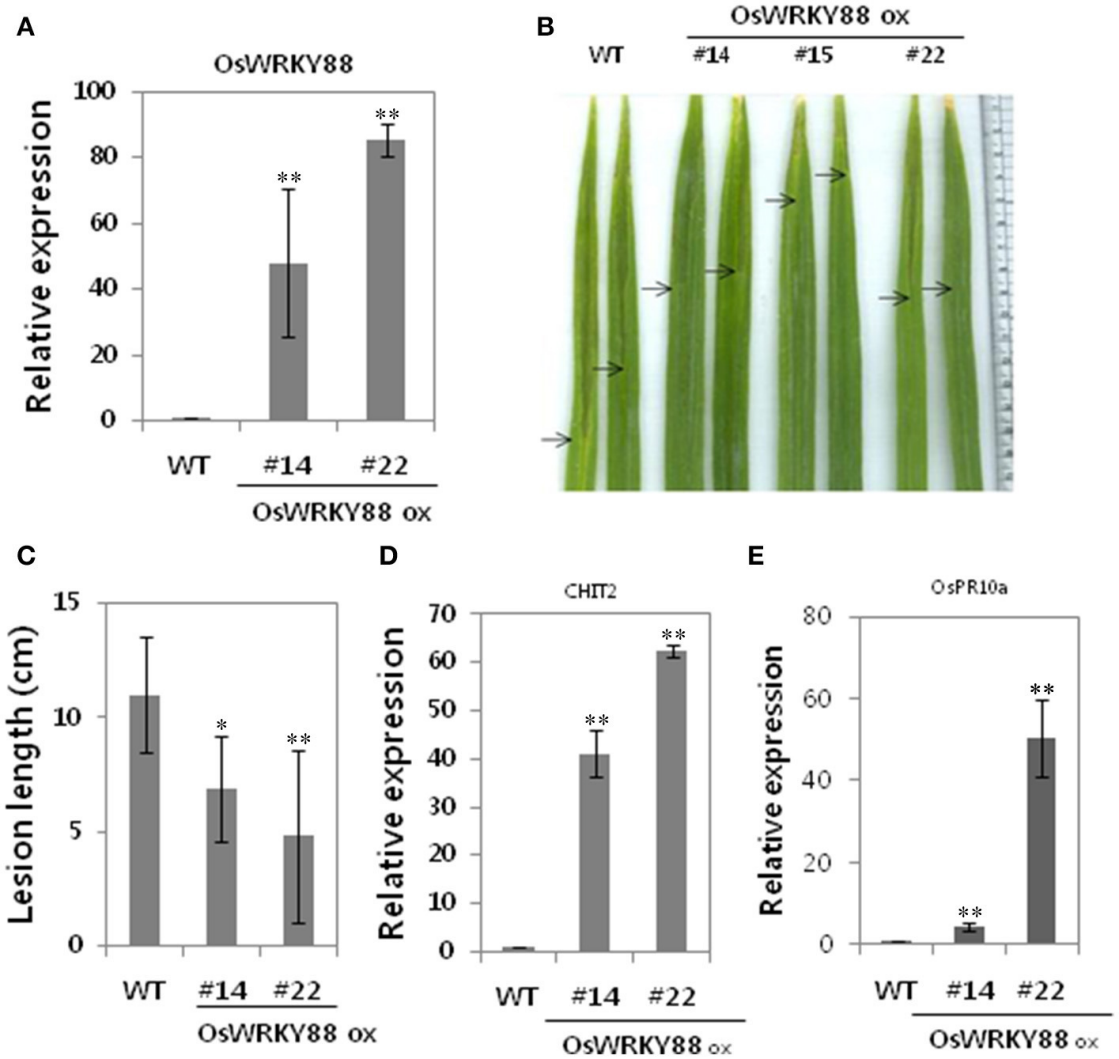
Analysis of *OsWRKY88* ox plants. **(A)** Expression analysis of *OsWRKY88* in *OsWRKY88* ox rice lines and the WT (Ilmi cultivar carrying Xa1). **(B,C)** *OsWRKY88* ox rice plants derived from the Ilmi cultivar were challenged with a compatible strain of *Xoo*. Photos were taken and lesion lengths were measured at 14 dpi. **(D,E)** Expression analysis of defense related genes (*OsPR10a, CHIT2*) in *OsWRKY88* ox lines. Asterisks indicate significant differences (^**^P < 0.01; ^*^P < 0.05).

We showed that six *OsWRKY* TFs were induced as part of SA-dependent Xa1-mediated resistance. Among them the transgenic plants overexpressing *OsWRKY88* were generated and analyzed. We found that *OsWRKY88* transcript levels were higher in #22 lines than that in #14 lines (Figure 5A). *OsWRKY88* ox lines were challenged with the compatible *Xoo* strain KACC10859 by the leaf clip method. After challenge, *OsWRKY 88* ox lines #14 and #22 exhibited shorter lesion lengths (~4 cm) than those (~12c m) of WT plants (Figures 5B,C), suggesting that *OsWRKY88* plays a role in *Xoo* resistance. We also performed qRT-PCR of defense related genes such as *CHIT2* and *OsPR10a*. Unlike in WT plants, *CHIT2* and *OsPR10a* were constitutively expressed in *OsWRKY88* ox lines #14 and #22 (Figures 5D,E), indicating that *Os*WRKY88 positively regulates the defense response to *Xoo*.

### OsWRKY88 directly regulates the *OsPR10a* promoter but not the *CHIT2* promoter

**Figure 6:**
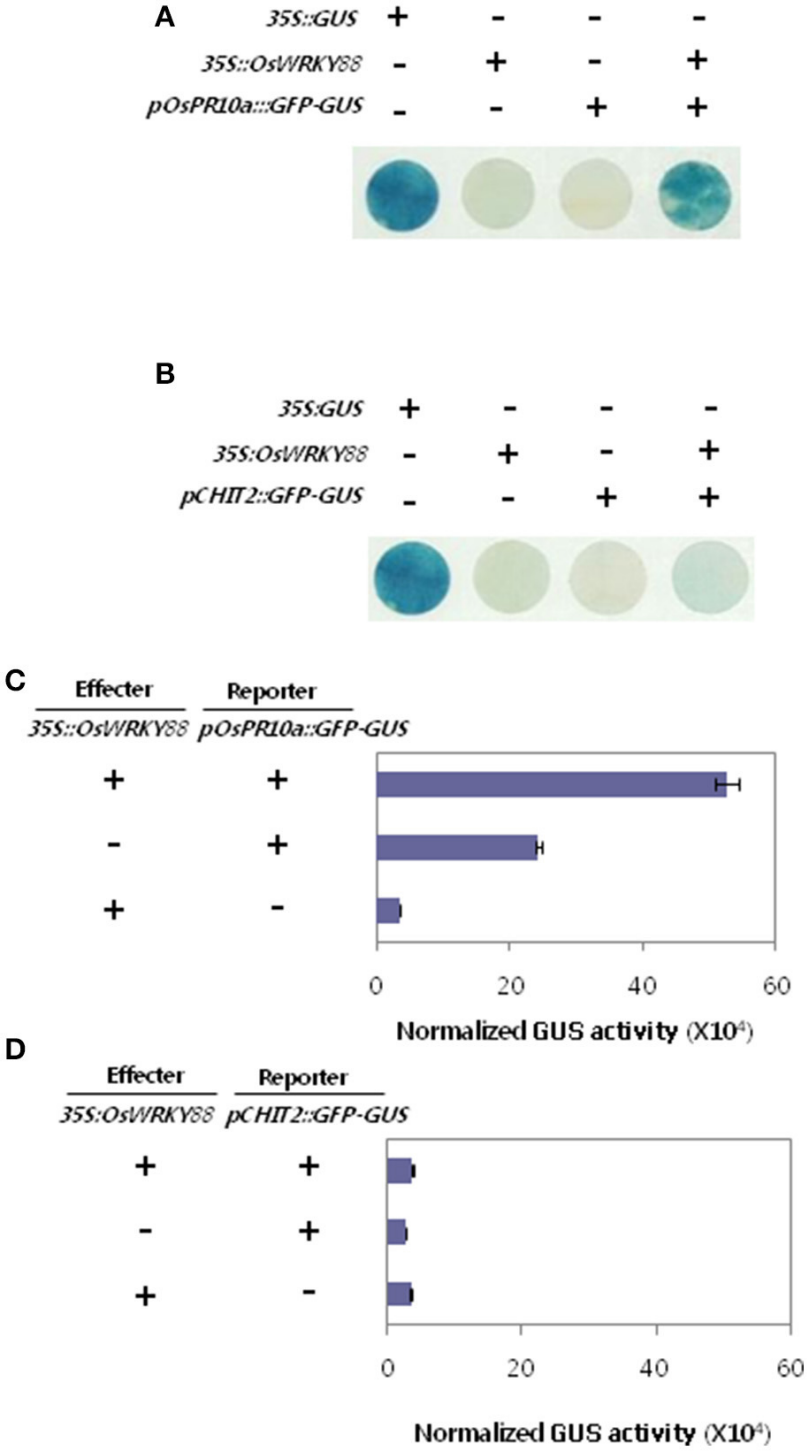
Promoter transient assays in *N. benthamiana*. **(A,B)** Agrobacterium carrying promoter-reporter constructs *pOsPR10a::GFP-GUS* or *pCHIT2::GFP-GUS* were co-infiltrated into leaves along with Agrobacterium carrying an effector construct (35S::OsWRKY88). Infiltrated leaves were removed at 24 hpi and visualized by GUS staining. **(C,D)** Infiltrated leaves used to measure GUS enzyme activity.

Defense related genes *OsPR10a* and *CHIT2* were expressed at a high level in OsWRKY88 ox lines. To examine whether OsWRKY88 directly regulates *OsPR10a* and *CHIT2*, we examined its effect on transcription from the *OsPR10a* and *CHIT2* gene promoters using the promoter transient assay (Figure 6). *Agrobacterium-*mediated transient assays for *pOsPR10a::GFP-GUS* (Hwang et al., 2008) were performed in *N. benthamiana* leaves (Figures 6A,C). The trans-activation activity of OsWRKY88 at the *OsPR10a* promoter was assessed using GUS staining and GUS enzyme activity. GUS activity was stronger in leaves co-infiltrated with *pOsPR10a::GFP-GUS* and *35S::OsWRKY88* than in leaves infiltrated with either *pOsPR10a::GFP-GUS* or *35S::OsWRKY88*. We also performed a CHIT2 promoter transient assay (Figures 6B,D), which revealed no significant difference in GUS staining and GUS enzyme activity between the sample co-infiltrated with a mixture of *pCHIT2::GFP-GUS* and *35S::OsWRKY88* and infiltrated with either *pCHIT2::GFP-GUS* or *35S::OsWRKY88*. These results suggest that *Os*WRKY88 trans-activates the *OsPR10a* promoter but not the *CHIT2* promoter.

## Discussion

Plants have developed unique immune systems for defense against a range of stresses. WRKY TFs have roles in biological processes such as growth, development, and responses to abiotic stress, but are also involved in regulating plant immune responses (Jimmy and Babu, 2015). In this study, expression of *OsWRKY* TF genes was profiled during pathogenic challenge with *Xoo* to elucidate the roles of *OsWRKY* TFs in the defense response.

Genes encoding WRKY superfamily TFs in *Arabidopsis* and rice were previously expression profiled in response to biotic stresses (Dong et al., 2003; Ryu et al., 2006). In this study, gene expression was modulated in 106 of the 111 *OsWRKY* TF genes examined in response to *Xoo* infection (Table 1). Approximately 96% of *OsWRKY* TFs were up- or down-regulated upon *Xoo* infection. The *OsWRKY* TF genes showed 8 different types of expression profiles (Table 1). Genes in type I, II, III, IV, and V (81 *OsWRKY* TF genes in total; ~73%of the 111 *OsWRKY* TF genes) were up-regulated after challenge with a compatible or an incompatible race of *Xoo*. In *Arabidopsis* leaves, gene expression was modulated in 49 of 53 *AtWRKY* TF genes (~90%) as assessed by northern blotting (Dong et al., 2003). Of these, 43 *AtWRKY* TF genes (~81%) were up-regulated after challenge with an incompatible pathogen. Research in rice reported that 9 of 15 (60%) *OsWRKY* TF genes tested were induced by an incompatible race of *Xoo* (Ryu et al., 2006). However, the study examined only 15 of the 125 known *OsWRKY* TF genes. In this study, 66 of 111 *OsWRKY* TF genes (type I, IV, and VII; 60%) were up-regulated by an incompatible race of *Xoo*. This discrepancy may be due to the sensitivity of methods such as RT-PCR and qRT-PCR when used for expression analysis. The previous study noted that *OsWRKY7, 10, 11, 30, 32, 67, 70, 83* (renamed *94* by CGSNL), and *85* (renamed *96* by CGSNL) were induced by an incompatible race of *Xoo* (Ryu et al., 2006). Of these, *OsWRKY7, 10, 11, 30, 32, 70, 94*, and *96* were also induced by an incompatible race of *Xoo* in our results. However, in contrast with the previous study (Ryu et al., 2006), our results showed clear down-regulation of *OsWRKY67* after exposure to incompatible and compatible *Xoo* races. This discrepancy is also predicted to be caused by the different methods used for expression analysis of *OsWRKY* TF genes in the two studies. Consistent with our data, previous studies showed that *OsWRKY03* (renamed *12* by CGSNL), *6, 13, 30, 45*, and *76* were induced by *Xoo* and were involved in regulation of *Xoo*-mediated resistance (Liu et al., 2005, 2007; Chujo et al., 2008; Qiu and Yu, 2009; Tao et al., 2009; Hwang et al., 2011; Han et al., 2013; Lee et al., 2013; Choi et al., 2015).

Studies exploring *OsWRKY* TF gene expression after infection with a compatible race of *Xoo* are scarce. Expression profiles of defense-related genes induced by compatible and incompatible pathogens have been identified (Jimmy and Babu, 2015). However, to our knowledge, the expression profiles of genes induced only by compatible pathogens have not been published. In this study, *OsWRKY* TF gene expression profiles were generated in response to challenge with a compatible race of *Xoo*. Sixteen *OsWRKY* TF genes were induced by compatible infection. Of these, 14 OsWRKY TFs (*OsWRKY4, 5, 14, 19, 28, 48, 78, 79, 82, 85, 86, 98, 101* and *121*) were down-regulated by incompatible infection, suggesting that some OsWRKY TFs might function as negative regulators in the defense response, resulting in increased disease susceptibility to *Xoo*. Previous research indicated that OsWRKY4 enhanced resistance to a necrotrophic pathogen, *Rhizotonia solani*, and was involved in the jasmonic acid (JA)-mediated pathway (Wang H. et al., 2015). This suggests that OsWRKY4 has a negative role in responding to challenge with biotrophic pathogens such as *Xoo*. Another TF, OsWRKY28, was found to act as a negative regulator in innate immunity, consistent with the gene expression profile seen in this study (Qiu et al., 2007). *Xoo* produces transcription activator-like (TAL) effectors that activate transcription of susceptibility (S) genes such as *OsSWEET14* and *OsTFX1* (Huang et al., 2016). Type II and III *OsWRKY* TFs might therefore be targets of TAL effectors.

Previous studies showed that SA was involved in *Xoo*-mediated resistance (Chujo et al., 2013; Choi et al., 2015). However, the incompatible interactions of *Xoo* with Mudanjiang 8 and Minghui 63 rice varieties were due to the Xa3/26 resistance factor, which is a typical pattern recognition receptor, rather than a NBS-LRR-type resistance factor. This resistance mechanism resembled pattern-triggered immunity, and it therefore remained unclear whether SA was involved in canonical R-gene-mediated resistance against *Xoo* in rice. In this study, we demonstrated that SA-deficient rice exhibited disease susceptibility when exposed to an incompatible race of *Xoo*, while the WT plant (Ilmi, carrying Xa1) exhibited hypersensitive-response-type resistance. This result suggested that SA was involved in Xa1-mediated resistance. *OsWRKY* TF genes induced during the Xa1-mediated resistance response were classified based on their expression profiles in response to SA. Of the 34 *OsWRKY* TF genes in Types I, III, and V, induction of 12 *OsWRKY* TF genes (*OsWRKY9, 11, 22, 23, 47, 58, 60, 64, 88, 106, 113, and 114*) by *Xoo* was compromised in SA-deficient rice lines generated by over-expression of *NahG*. Among them 6 *OsWRKY* TF genes (*OsWRKY11, 23, 47, 58, 60, 88*) were induced by SA. We therefore propose that Xa1-mediated resistance involves both SA-dependent and SA-independent pathways. Induction of *OsWRKY* TF genes such as *OsWRKY10, 30, 45, 62, 82* (renamed *89* by CGSNL), and *83* (renamed *94* by CGSNL) by SA was reported previously (Ryu et al., 2006). An additional study reported that *OsWRKY19, 45, 62*, and *76* were induced by the SA analog BTH (Shimono et al., 2007). Studies of individual *OsWRKY* TF genes indicated that *OsWRKY03* (renamed *12* by CGSNL), *OsWRKY6, OsWRKY33* (renamed *81* by CGSNL), *OsWRKY51*, and *OsWRKY77* were induced by SA (Liu et al., 2005; Koo et al., 2009; Hwang et al., 2011, 2016; Lan et al., 2013). However, 12 *OsWRKY* TF genes profiled in this study (*OsWRKY9, 11, 22, 23, 47, 58, 60, 64, 88, 106, 113*, and *114*) were not described previously, possibly because only *OsWRKY* TF genes involved in Xa1 mediated resistance were examined in this study. Overall, these results suggest that OsWRKY TFs regulate both SA-dependent and SA-independent Xa1-mediated resistance.

In this study, SA-deficient rice plants were generated by transforming rice plants with *NahG*, which degrades SA to catechol. However, several studies have questioned using *NahG* rice to study the effect of SA on plant resistance to infection (van Wees and Glazebrook, 2003; Yang et al., 2004; Mutuku et al., 2015). van Wees and Glazebrook (2003) arrived at false conclusions concerning the role of SA in plants defense because catechol affected non-host resistance in the *Arabidopsis* plants expressing *NahG*. Another study claimed that catechol had no effect on resistance to *S. hermonthica* (Mutuku et al., 2015). In this study, we demonstrated that catechol had no effect on Xa1-mediated resistance, confirming that the effects observed in SA-deficient rice were not due to catechol production. The effect of catechol on plant resistance would appear to depend on the pathosystem studied.

The function of *OsWRKY88*, a gene whose expression was altered during SA-dependent Xa1-mediated resistance, was analyzed. *OsWRKY88* ox lines showed increased resistance to *Xoo* and activation of the defense response. We demonstrated that OsWRKY88 directly activated the *OsPR10a* promoter but not the *CHIT2* promoter. The higher level of the *CHIT2* transcript might be due to the presence of another transcription factor induced by OsWRKY88. Some studies claim that transcription regulatory cascades of TFs are required for disease resistance (Cheng et al., 2015). *OsWRKY22* and *OsWRKY23* (previously known as *OsWRKY31*) have been previously reported to be positive regulators of resistance to *M. oryzae*, although the effect of *OsWRKY22* and *OsWRKY23* on *Xoo* infection was not investigated (Zhang et al., 2008; Abbruscato et al., 2012). Expression profiling of *OsWRKY* TF genes was performed after the infection of rice with *Xoo*, which revealed changes in expression of most of the genes. *OsWRKY* TF gene expression in Xa1-mediated resistance appeared to involve both SA-dependent and SA-independent pathways. Therefore, we propose that *OsWRKY* TF genes are involved in Xa1-mediated resistance.

## Author contributions

NC and DH wrote the manuscript. DH designed the experiments. NC, SL, and CC performed qRT-PCR. EL analyzed the transgenic plants. SP, IA, and SB identified database *OsWRKY* TF gene sequences and designed primers. CH critically revised the manuscript.

### Conflict of interest statement

The authors declare that the research was conducted in the absence of any commercial or financial relationships that could be construed as a potential conflict of interest.

## References

[B1] Abbruscato P. Nepusz T. Mizzi L. Del Corvo M. Morandini P. Fumasoni I.. (2012). OsWRKY22, a monocot WRKY gene, plays a role in the resistance response to blast. Mol. Plant Pathol. 13, 828–841. 10.1111/j.1364-3703.2012.00795.x 22443363 PMC6638809

[B2] Bagnaresi P. Biselli C. Orrù L. Urso S. Crispino L. Abbruscato P.. (2012). Comparative transcriptome profiling of the early response to *Magnaporthe oryzae* in durable resistant vs susceptible rice (*Oryza sativa* L.) genotypes. PLoS ONE 7:e51609. 10.1371/journal.pone.0051609 23251593 PMC3520944

[B3] Berri S. Abbruscato P. Faivre-Rampant O. Brasileiro A. C. Fumasoni I. Satoh K.. (2009). Characterization of WRKY co-regulatory networks in rice and *Arabidopsis*. BMC Plant Biol. 9, 186–120. 10.1186/1471-2229-9-120 19772648 PMC2761919

[B4] Cai M. Qiu D. Yuan T. Ding X. Li H. Duan L.. (2008). Identification of novel pathogen-responsive cis-elements and their binding proteins in the promoter of OsWRKY13, a gene regulating rice disease resistance. Plant Cell Environ. 31, 86–96. 10.1111/j.1365-3040.2007.01739.x 17986178

[B5] Cheng H. Liu H. Deng Y. Xiao J. Li X. Wang S. (2015). The WRKY45-2 WRKY13 WRKY42 transcriptional regulatory cascade is required for rice resistance to fungal pathogen. Plant Physiol. 167, 1087–1099. 10.1104/pp.114.256016 25624395 PMC4348788

[B6] Choi C. Hwang S. Fang I. Kwon S. Park S. Ahn I.. (2015). Molecular characterization of *Oryza sativa* WRKY6, which binds to W-box-like element 1 of the *Oryza sativa* pathogenesis-related (PR) 10a promoter and confers reduced susceptibility to pathogens. New Phytol. 208, 846–859. 10.1111/nph.13516 26083148

[B7] Chu Z. Yuan M. Yao J. Ge X. Yuan B. Xu C.. (2006). Promoter mutations of an essential gene for pollen development result in disease resistance in rice. Genes Dev. 20, 1250–1255. 10.1101/gad.1416306 16648463 PMC1472899

[B8] Chujo T. Kato T. Yamada K. Takai R. Akimoto-Tomiyama C. Minami E.. (2008). Characterization of an elicitor-induced rice WRKY gene, *OsWRKY71*. Biosci. Biotechnol. Biochem. 72, 240–245. 10.1271/bbb.70553 18175928

[B9] Chujo T. Miyamoto K. Shimogawa T. Shimizu T. Otake Y. Yokotani N.. (2013). OsWRKY28, a PAMP-responsive transrepressor, negatively regulates innate immune responses in rice against rice blast fungus. Plant Mol. Biol. 82, 23–37. 10.1007/s11103-013-0032-5 23462973

[B10] Ciolkowski I. Wanke D. Birkenbihl R. P. Somssich I. E. (2008). Studies on DNA-binding selectivity of WRKY transcription factors lend structural clues into WRKY-domain function. Plant Mol. Biol. 68, 81–92. 10.1007/s11103-008-9353-1 18523729 PMC2493524

[B11] Delaney T. P. Ukness S. Vernooij B. Friedrich L. Weymann K. Negrotto D.. (1994). A central role of salicylic acid in plant disease resistance. Science 266, 1247–1250. 10.1126/science.266.5188.1247 17810266

[B12] Dong J. Chen C. Chen Z. (2003). Expression profiles of the *Arabidopsis* WRKY gene superfamily during plant defense response. Plant Mol. Biol. 51, 21–37. 10.1023/A:1020780022549 12602888

[B13] Eulgem T. Rushton P. J. Robatzek S. Somssich I. E. (2000). The WRKY superfamily of plant transcription factors. Trends Plant Sci. 5, 199–206. 10.1016/S1360-1385(00)01600-9 10785665

[B14] Gu K. Y. Yang B. Tian D. S. Wu L. F. Wang D. J. Sreekala C.. (2005). R gene expression induced by a type-III effector triggers disease resistance in rice. Nature 435, 1122–1125. 10.1038/nature03630 15973413

[B15] Han M. Ryu H. S. Kim C. Y. Park D. S. Ahn Y. K. Jeon J. S. (2013). OsWRKY30 is a transcription activator that enhances rice resistance to the *Xanthomonas oryzae* pathovar *oryzae*. J. Plant Biol. 56, 258–265. 10.1007/s12374-013-0160-0

[B16] Huang S. Antony G. Li T. Liu B. Obasa B. Yang B.. (2016). The broadly effective recessive resistance gene xa5 of rice is a virulence effector-dependent quantitative trait for bacterial blight. Plant J. 86, 186–194. 10.1111/tpj.13164 26991395

[B17] Hwang S. H. Kwon S. I. Jang J. Y. Fang I. L. Lee H. Choi C.. (2016). OsWRKY51, a rice transcription factor, functions as a positive regulator in defense response against *Xanthomonas oryzae* pv. oryzae Plant Cell Rep. 35, 1975–1985. 10.1007/s00299-016-2012-0 27300023

[B18] Hwang S. H. Lee I. A. Yie S. W. Hwang D. J. (2008). Identification of an OsPR10a promoter region responsive to salicylic acid. Planta 227, 1141–1150. 10.1007/s00425-007-0687-8 18193274 PMC2270913

[B19] Hwang S. H. Yie S. W. Hwang D. J. (2011). Heterologous expression of OsWRKY6 gene in *Arabidopsis* activates the expression of defense related genes and enhances resistance to pathogens. Plant Sci. 181, 316–323. 10.1016/j.plantsci.2011.06.007 21763543

[B20] Iyer A. S. McCouch S. R. (2004). The rice bacterial blight resistance gene *xa5* encodes a novel form of disease resistance. Mol. Plant Microbe Interact. 17, 1348–1354. 10.1094/MPMI.2004.17.12.1348 15597740

[B21] Jimmy J. L. Babu S. (2015). Role of OsWRKY transcription factors in rice disease resistance. Trop. Plant Pathol. 40, 355–361. 10.1007/s40858-015-0058-0

[B22] Karimi M. Inze D. Depicker A. (2002). GATEWAY vectors for *Agrobacterium*-mediated plant transformation. Trends Plant Sci. 7, 193–195. 10.1016/S1360-1385(02)02251-3 11992820

[B23] Kauffman H. E. Reddy A. P. K. Hsieh S. P. Y. Merca S. D. (1973). An improved technique for evaluating resistance of rice varieties to *Xanthomonas oryzae*. Plant Dis. Rep. 57, 537–541.

[B24] Kim D. Y. Kwon S. I. Choi C. Lee H. Ahn I. Park S. R.. (2013). Expression analysis of rice VQ genes in response to biotic and abiotic stresses. Gene 529, 208–214. 10.1016/j.gene.2013.08.023 23958655

[B25] Kim Y. H. Park H. M. Choi M. S. Yun H. T. Choi I. S. Shin D. B. (2009). The effects of co-cultivation medium and culture conditions on rice transformation efficiency. Korean J. Breed. Sci. 41, 252–260. 10.1186/1746-4811-7-49

[B26] Koo S. C. Moon B. C. Kim J. K. Kim C. Y. Sung S. J. Kim M. C.. (2009). OsBWMK1 mediates SA-dependent defense responses by activating the transcription factor OsWRKY33. Biochem. Biophys. Res. Commun. 387, 365–370. 10.1016/j.bbrc.2009.07.026 19607808

[B27] Lan A. Huang J. Zhao W. Peng Y. Chen Z. Kang D. A. (2013). Salicylic acid-induced rice (*Oryza sativa* L.) transcription factor OsWRKY77 is involved in disease resistance of *Arabidopsis thaliana*. Plant Biol. 15, 452–461. 10.1111/j.1438-8677.2012.00664.x 23061987

[B28] Lee H. Ko Y. J. Cha J. Y. Park S. R. Ahn I. Hwang D. J. (2013). The C-terminal region of OsWRKY30 is sufficient to confer enhanced resistance to pathogen and activate the expression of defense-related genes. Plant Biotechnol. Rep. 18, 1–10. 10.1007/s11816-012-0252-1

[B29] Li X. (2011). Infiltration of *Nicotiana* b*enthamiana* protocol for transient expression via Agrobacterium. Bioprotocol 1, 1–3. 10.21769/BioProtoc.95

[B30] Liu Q. Yuan M. Zhou Y. Li X. Xiao J. Wang S. (2011). A paralog of the MtN3/saliva family recessively confers race-specific resistance to *Xanthomonas oryzae* in rice. Plant Cell Environ. 34, 1958–1969. 10.1111/j.1365-3040.2011.02391.x 21726237

[B31] Liu X. Q. Bai X. Q. Qian Q. Wang X. J. Chen M. S. Chu C. C. (2005). OsWRKY03, a rice transcriptional activator that functions in defense signaling pathway upstream of OsNPR1. Cell Res. 15, 593–603. 10.1038/sj.cr.7290329 16117849

[B32] Liu X. Bai X. Wang X. Chu C. (2007). OsWRKY71, a rice transcription factor, is involved in rice defense response. J. Plant Physiol. 164, 969–979. 10.1016/j.jplph.2006.07.006 16919842

[B33] Mutuku J. M. Yoshida S. Shimizu T. Ichihashi Y. Wakatake T. Takahashi A.. (2015). The WRKY45-dependent signaling pathway is required for resistance against *Striga hermonthica* parasitism. Plant Physiol. 168, 1152–1163. 10.1104/pp.114.256404 26025049 PMC4741350

[B34] Pandey S. P. Somssich I. E. (2009). The role of WRKY transcription factors in plant immunity. Plant Physiol. 150, 1648–1655. 10.1104/pp.109.138990 19420325 PMC2719123

[B35] Qiu D. Xiao J. Ding X. Xiong M. Cai M. Cao Y.. (2007). OsWRKY13 mediates rice disease resistance by regulating defense-related genes in salicylate- and jasmonate-dependent signaling. Mol. Plant Microbe Interact. 20, 492–499. 10.1094/MPMI-20-5-0492 17506327

[B36] Qiu Y. Yu D. (2009). Over-expression of the stress-induced OsWRKY45 enhances disease resistance and drought tolerance in *Arabidopsis*. Environ. Exp. Bot. 65, 35–47. 10.1016/j.envexpbot.2008.07.002

[B37] Rice WRKY Working Group. (2012). Nomenclature report on rice WRKY's – conflict regarding gene names and its solution. Rice 5, 3–5. 10.1186/1939-8433-5-3 24764503 PMC3834489

[B38] Ryu H. S. Han M. Lee S. K. Cho J. I. Ryoo N. Heu S.. (2006). A comprehensive expression analysis of the WRKY gene superfamily in rice plants during defense response. Plant Cell Rep. 25, 836–847. 10.1007/s00299-006-0138-1 16528562

[B39] Schmittgen T. D. Livak K. J. (2008). Analyzing real-time PCR data by the comparative C_*T*_ method. Nat. Protoc. 3, 1101–1108. 10.1038/nprot.2008.73 18546601

[B40] Shimono M. Sugano S. Nakayama A. Jiang C. J. Ono K. Toki S.. (2007). Rice WRKY45 plays a crucial role in benzothiadiazole-inducible blast resistance. Plant Cell 19, 2064–2076. 10.1105/tpc.106.046250 17601827 PMC1955718

[B41] Song W. Y. Wang G. L. Chen L. Kim K. S. Holstein T. Wang B.. (1995). A receptor kinase-like protein encoded by the rice disease resistance gene, Xa21. Science 270, 1804–1806. 10.1126/science.270.5243.1804 8525370

[B42] Sun C. Palmqvist S. Olsson H. Borén M. Ahlandsberg S. Jansson C. (2003). A novel WRKY transcription factor, SUSIBA2, participates in sugar signaling in barley by binding to the sugar-responsive elements of the iso1 promoter. Plant Cell 15, 2076–2092. 10.1105/tpc.014597 12953112 PMC181332

[B43] Sun X. Cao Y. Yang Z. Xu C. Li X. Wang S.. (2004). *Xa26*, a gene conferring resistance to *Xanthomonas oryzae* pv. *oryzae* in rice, encodes an LRR receptor kinase-like protein. Plant J. 37, 517–527. 10.1046/j.1365-313X.2003.01976.x 14756760

[B44] Tao Z. Liu H. Qiu D. Zhou Y. Li X. Xu C.. (2009). A Pair of allelic WRKY genes play opposite roles in rice-bacteria interactions. Plant Physiol. 151, 936–948. 10.1104/pp.109.145623 19700558 PMC2754648

[B45] Tian D. Wang J. Zeng X. Gu K. Qiu C. Yang X.. (2014). The rice TAL effector-dependent resistance protein *XA10* triggers cell death and calcium depletion in the endoplasmic reticulum. Plant Cell 26, 497–515. 10.1105/tpc.113.119255 24488961 PMC3963592

[B46] van Wees S. C. Glazebrook J. (2003). Loss of non-host resistance of *Arabidopsis* NahG to *Pseudomonas syringae* pv. *phaseolicola* is due to degradation products of salicylic acid. Plant J. 33, 733–742. 10.1046/j.1365-313X.2003.01665.x 12609045

[B47] Wang C. Zhang X. Fan Y. Gao Y. Zhu Q. Zheng C.. (2015). XA23 is an executor R protein and confers broad-spectrum disease resistance in rice. Mol. Plant 8, 290–302. 10.1016/j.molp.2014.10.010 25616388

[B48] Wang H. Meng J. Peng X. Tang X. Zhou P. Xiang J.. (2015). Rice WRKY4 acts as a transcriptional activator mediating defense responses toward *Rhizoctonia solani*, the causing agent of rice sheath blight. Plant Mol. Biol. 89, 157–171. 10.1007/s11103-015-0360-8 26275661

[B49] Wei T. Ou B. Li J. Zhao Y. Guo D. Zhu Y.. (2013). Transcriptional profiling of rice early response to *Magnaporthe oryzae* identified OsWRKYs as important regulators in rice blast resistance. PLoS ONE 8:e59720. 10.1371/journal.pone.0059720 23544090 PMC3609760

[B50] Yang Y. Qi M. Mei C. (2004). Endogenous salicylic acid protects rice plants from oxidative damage caused by aging as well as biotic and abiotic stress. Plant J. 40, 909–919. 10.1111/j.1365-313X.2004.02267.x 15584956

[B51] Yoshimura S. Yamanouchi U. Katayose Y. Toki S. Wang Z. X. Kono I.. (1998). Expression of *Xa1*, a bacterial blight-resistance gene in rice, is induced by bacterial inoculation. Proc. Natl. Acad. Sci. U.S.A. 95, 1663–1668. 10.1073/pnas.95.4.1663 9465073 PMC19140

[B52] Zhang J. Peng Y. Guo Z. (2008). Constitutive expression of pathogen-inducible *OsWRKY31* enhances disease resistance and affects root growth and auxin response in transgenic rice plants. Cell Res. 18, 508–521. 10.1038/cr.2007.104 18071364

[B53] Zhang Y. J. Wang L. J. (2005). The WRKY transcription factor superfamily: its origin in eukaryotes and expansion in plants. BMC Evol. Biol. 5:1. 10.1186/1471-2148-5-1 15629062 PMC544883

