# Supplementary material for: Genome-Wide Expression Profiling of OsWRKY Superfamily Genes during Infection with Xanthomonas oryzae pv. oryzae Using Real-Time PCR
Source: Front Plant Sci. 2017 Sep 20;8:1628. doi: 10.3389/fpls.2017.01628 (PMC5611491; doi:10.3389/fpls.2017.01628)
Supplement: Supplementary file 1 [file Presentation1.PDF]

Table S1. List of qRT-PCR primers for *OsWRKY* TFs expression profiling.

Gene #	Locus #	Forward primer	Reverse primer
OsWRKY01	LOC_Os01g14440	GCAGGTGCTGTACAACCAGT	CTGACGGTGTCGGAGAGAG
OsWRKY02	LOC_Os10g42850	ATGCTCGTCGTCACCTACAC	GGCGTATGTTGGATGAGTTG
OsWRKY03	LOC_Os03g55080	ACAACGCCCTGGCTATAATAA	CTCCAGGAGGCACAATATCA
OsWRKY04	LOC_Os03g55164	GAGCAAAGTCACCGGAGGAA	TTGGGATTGGACTGACTCGC
OsWRKY05	LOC_Os05g04640	ATGATGGTGCAGAAGCAACG	TGCTGACCATAAATCCGGC
OsWRKY06	LOC_Os03g58420	GACGCTCTTGGATTTGAACA	TGCTGGAACTGGATGTGATT
OsWRKY07	LOC_Os05g46020	TTCCGGACGAAGTCAGAGAT	ACGGACTTCTTGCCGTACTT
OsWRKY08	LOC_Os05g50610	GTACACCCGGCTCAAGATGT	GAGGATTTCGAGGATCAAGC
OsWRKY09	LOC_Os01g18584	TCACAGAGACATACGTGACA	CAAGTATGTGTGGGCGTCAT
OsWRKY10	LOC_Os01g09100	CCTTTGGTGAATTCCTGGAG	GCCATACTGATCATTGTTCCC
OsWRKY11	LOC_Os01g43650	AGCCCAAGAAGAAAGCTGAG	TATGGGCTGTTCTTGACTGC
OsWRKY12	LOC_Os01g43550	CGACCTCGAGTACCTGGATT	GATCATCACCTCGTGTCTGC
OsWRKY13	LOC_Os01g54600	AGGAATCTGAGCCGGTTG	TCAACCACAGCCTTCTGTTC
OsWRKY14	LOC_Os01g53040	AAGTGGAACCCGAGGAAAC	CGTAGAGCCACCGGAACT
OsWRKY15	LOC_Os01g46800	GATGTCGGACTGCCAGATTA	GAGTCCAGCGAGAACATGAA
OsWRKY16	LOC_Os01g47560	ACCACGGCATGACTACGAT	GCTGATTGCACTGTTAAGGC
OsWRKY17	LOC_Os01g74140	CTGCACCTCTGACAACGAGT	TGCAGGGAGACATGGGAGTA
OsWRKY18	LOC_Os10g18099	TTGATGGACTCAGGCAGATG	ATTCGCACGTATCCAAATCA
OsWRKY19	LOC_Os05g49620	AACTCCGACTGCCAGTTCA	CTCGTGGTCCATGTCAACC
OsWRKY20	LOC_Os01g60540	CCGTCGATGTTAGCAGAGAG	TGCAAGGCTTCAGCATGTA
OsWRKY21	LOC_Os01g60640	CATAGAGCAGCTGTACCGGG	TCTCTGCTAACATCGACGGC
OsWRKY22	LOC_Os01g60490	ACAGGTGCACGTACCATCAG	TTGCATGGAGGAAGCCATGT
OsWRKY23	LOC_Os01g53260	GGCTACCGATGGAGGAAGTA	ACGTAGTCACCACGATGCTC
OsWRKY25	LOC_Os08g13840.2	CGTCGTCGTCTCCTGTCTC	GATTCGTGAAGTCCAGCGT
OsWRKY26	LOC_Os01g51690	TACGTGGTGACGATGTACGA	AAGGTCTGGGTGACTGATCC
OsWRKY27	LOC_Os01g40430	GAGGCCGGGTTATTCCTGTC	TTCGTCTTTCTTGCTGGCGT
OsWRKY28	LOC_Os06g44010	TCGTGGATTGAGCAGACTTC	TTCTCCTCCACGAGAACCTT
OsWRKY29	LOC_Os07g02060	ACCTACGAGGGCCAACACT	TGCAGGTTGCTGTACAGTTG
OsWRKY30	LOC_Os08g38990	TGGGCAGAAAGTTGTCAAAG	TACGTCGTGATGACGGATTT
OsWRKY31	LOC_Os06g30860	GTCGGCGAGGAGAAAGTATG	CAGCGAGCGAATCCACATTA
OsWRKY32	LOC_Os02g53100	GAGGGCCCTAAAGAGGAAAC	TCCTCGATCATCTCTGCTTG
OsWRKY34	LOC_Os02g43560	GTGCTGGATGACGGGTACAA	TGAGCATATGTACCTGGGATGG
OsWRKY36	LOC_Os04g46060	GCGAGAGTGGATAGCGAGAT	CCCTTCCACCATGAATTAGG
OsWRKY37	LOC_Os04g50920	TATCAAGGGCTCACCCTACC	AGCATGTTTGGGTCAGTACG
OsWRKY38		GGCTGGAGGATGCTGACAA	GTACTTCCTCCACCGGAAGC
OsWRKY39	LOC_Os02g16540	AGTACGGACAGAAGCCCATC	CGGTGTAGGTGAGGATGAAG
OsWRKY40	LOC_Os11g02530	AACTCCAAACACCCAAGGAG	GTGATCGCCATTGTAGCTGT

OsWRKY41	LOC_Os11g45924	TGAATCAATGGATAGCTGAAGG	ATGGACAGGCTGGATGATTT
OsWRKY42	LOC_Os02g26430	GCAGTCTGCTTCAGATTATGCT	TTTCTTGGAGCAGTGGCA
OsWRKY43	LOC_Os05g49210	CTGGCAGTAACCAAAGCTGA	CAGTGTGACGAACTGCATCT
OsWRKY45	LOC_Os05g25770	ATTCGGTGGTCGTCAAGAAC	ACAGCTGGTCGTACTTGTGC
OsWRKY46	LOC_Os11g02480	GGACGATTATGCAGAAGCAA	GATGCATCTTGACTGACCGA
OsWRKY47	LOC_Os07g48260	AGTCGACCCTCTTCGTCAGA	AGGACAGTACCGGAGAGTGG
OsWRKY48	LOC_Os05g40060	TCGTTTCTTGTTCGTCTCCG	GAACGAGCTGTCTCCAAAGT
OsWRKY49	LOC_Os05g49100	CCATCATCGCCTCTACCAC	TAATCCTGCGCCATGAAGT
OsWRKY50	LOC_Os11g02540	CTGCGAGCCACTCTACTACG	ATTATCATTGTCGCGGTTGA
OsWRKY51	LOC_Os04g21950	CTTATTCTGCAGCTGTCCCA	GAACTTGGACACCGTCATGT
OsWRKY52	LOC_Os11g02470	CTCCAGCGAACAGTAGTTCC	ACGACTTGTACTCCTCACCA
OsWRKY53	LOC_Os05g27730	GATCTCATCGCTTCTCAGCA	ACTTGCTGCTCTTGCTCCTT
OsWRKY54	LOC_Os05g40080	TGAGAGGAAAGCGACAATGG	AGTAAAACCTCGGGTGCTTG
OsWRKY55	LOC_Os03g20550	ATGTCTCCTGTGCCGAGTC	GGTATCCACCTTCCTCATGC
OsWRKY56	LOC_Os01g62514	CTGTGCTCGGTGTACTCAGG	TGCGGAAATACAGAGAGCTG
OsWRKY58	LOC_Os05g45230	ACGAGCTCCTTCTCTTCTCT	CCTGCCGAGGGGTATAAAAC
OsWRKY60	LOC_Os03g45450	GTACGGGCAGAAGTTCATCA	GTGCCACTCCACCTTCTTCT
OsWRKY62	LOC_Os09g25070	CTTACTTCCGCTGCGCATTC	GACGAATTCGGTTGTCTGCG
OsWRKY63	LOC_Os11g45920	AGAAATCAAGTCGGTGCGGT	ACTGGCAGAGACATGGTTGG
OsWRKY64	LOC_Os12g02450	TCTTCTGATTCTCCGCATGA	TCTCTTGTGTTGGCGAAGAG
OsWRKY65	LOC_Os12g02470	TAACGACGGTGCCAGATTT	GGCACCTTCAATTTGCTTCT
OsWRKY66	LOC_Os02g47060	GAGCCTGATCTTCTCCAAGG	AGACCAATCCAGCATGTACG
OsWRKY67	LOC_Os05g09020	GCTTCCGTAGGACTGAACCC	TGTAGCTGGCCATGAAAGGG
OsWRKY68	LOC_Os04g51560	TCCAAGAGAAGGAAGAACCG	CGATCTTCGAGCTGATTGC
OsWRKY69	LOC_Os08g29660	ACCTGCAGGAGGTCGTGT	CGAAGTCGAAGCAGTAGCTC
OsWRKY70	LOC_Os05g39720	ACTCTTACACGAGCCAGCAG	ACATGATGCCGTCGTCTCTC
OsWRKY71	LOC_Os02g08440	TCTCCAAGCTCTACGTCCAC	GGAGCAAATGAGCATCTGAA
OsWRKY72	LOC_Os11g29870	CACTTCATGAGCTCCTCTTCC	TCCATCCTAATTGACGACGA
OsWRKY73	LOC_Os06g05380	TAGGGTTTCAGTGAGAGCGA	CGCAATCTTCTGCCCATACT
OsWRKY74	LOC_Os09g16510	GGGCAGAAAGAGATTCTTGG	TAGATCACGTCGAACACCGT
OsWRKY75	LOC_Os05g25700	CCAGAACGACCCGGACTACC	ATGCGGAGGAGGTCCAACTG
OsWRKY76	LOC_Os09g25060	GTTATCGGGCAAGAAGAGGA	CTGATGCCTGTTGCTGTTG
OsWRKY77	LOC_Os01g40260	GTCCAGCTACCTCTCCTTCG	TTGTGCTGCTTTGAGTGGA
OsWRKY78	NM_188117	GTGGTGTGTGCTTAGAGGCT	TGCCACTGTGATTTGCGTTG
OsWRKY79	LOC_Os03g21710	TACTACGGCGAGCACACCT	GCGAAGCTGATGATGTTGTT
OsWRKY80	LOC_Os03g63810	ATATGCCCGTTCTCATCACA	GGAGGAAGAAGACGATGAGC
OsWRKY81	LOC_Os03g33012	TTCTCGTCGACAACGACTTC	GGGATGGCAGAGTTGGTATT
OsWRKY82	LOC_Os05g14370	GGATGAACACATGCACAAGC	CATCAATCTCCCAGCTGTCC
OsWRKY83	LOC_Os05g50680	GGCTCTTGTTCACATCTCCA	CTGATGACAGCCTTCACGTC
OsWRKY84	LOC_Os05g40070	GTGCTCGACTTCACAAAAGC	CCTCTTCCTTCAAGATCGGC
OsWRKY85	NM_185588	CACACCTGCCACCAGAAGCA	CCGTGCCTGAAGTTGACGAG

OsWRKY86	NM_185324	GACGACTGGGATTGCCTCAA	GTGCATGCAGGCCTTGGAG
OsWRKY87	LOC_Os07g39480	TTCAAGGTTGAACCCTCTCC	TCATGGGTGCTACCAGATGT
OsWRKY88	LOC_Os07g40570	GTCAGAAGGCAGTGAAGGGT	CTGCAGAGTGCTCCACATTT
OsWRKY89	LOC_Os08g17400	AGCGATCACCTTCAGGAACT	CAGTTGAAGCCATCACCAAC
OsWRKY90	LOC_Os09g30400	CAGCTGCCAGAAATAGTGGA	GAGAACGAAGGAGCTGAAGG
OsWRKY93	AC123514	TCCATTCCTCGAAGCATATTGGT	CATCTCCCCTGCATGTGTGT
OsWRKY94	LOC_Os12g40570	ATTAAAGTGCCCGCCATTAG	GAACCCTTAATCGGCTTCTG
OsWRKY95	LOC_Os12g02440	CTGCGTCCACACCTATTCAC	CCACCACTGATGTCGATGAT
OsWRKY96	LOC_Os12g32250	CAAGCCATTCTCCACTCAGA	ACCCATGACCATGTCTGAAA
OsWRKY97	LOC_Os12g02420	ACGATTATGCGGAAGCAAGT	GATGCATCTTGACTGACCGA
OsWRKY98	NM_190356	GCCTCTCCTTACGACGATGG	AGCTCCTTGGGAAGTTGGTG
OsWRKY100	NM_189575	GCTCGACGACGGCTACAAGT	GCTTCTTCACCCGGCAGTTG
OsWRKY101	NM_189614	CGGTGAGGGACGAGAAGAAA	ATCTCTCTGCGTCGATGGTG
OsWRKY102	LOC_Os01g08710	CGGTGAGGATGAAGAAGGTT	GTGCACCGGTAATAGCTCCT
OsWRKY104	LOC_Os11g02520	CGAATCTTTTGGTCATAGTGACTG	TTGTTCATTGTCGCTCGGAA
OsWRKY105	LOC_Os08g09800	CAGTCAGGTCAAGCTCAGGA	CATACGGCTGCGTGTTAAAT
OsWRKY106	LOC_Os08g09810	ATGCTCTGCACTTTGTCTGG	TCTTCAGGGTTTGGGAACTC
OsWRKY107	LOC_Os01g09080	CAGCCATACTACGCCAAGG	ATGGCTCTCTGCAGGTACG
OsWRKY108	LOC_Os01g60600	ATGGGTACCAGTGGAGGAAG	CGGTGGTAACTGCACCTGTA
OsWRKY109	LOC_Os05g03900	GGAGGAAATATGGGCAGAAA	ACAAGAACATTCGGTGCAGT
OsWRKY110	AP008213	TTGAAGGTGCCTTGTACCCC	GATGACTGGAGGCAGGGATG
OsWRKY111	LOC_Os05g50700	ACCATCACACACGCTAGCTC	GAGATCCCAATCGTTGGAGTA
OsWRKY112	LOC_Os09g09630	GTTGCATTGACCACCGAATG	GCACGTTGTTGAATCGAAGG
OsWRKY113	LOC_Os06g06360	CAGAGTGCCAGATGCAAGTT	ACCGAGGATGTCCTTCTGAC
OsWRKY114	LOC_Os12g02400	CACCCATGTTCCAAGTGACA	ATCGTCAGGGTGACCATTTG
OsWRKY115	LOC_Os07g27670	AGCAGAAGCTGGAGGAACAT	AGGGTATGAACAGCCACGAC
OsWRKY117	LOC_Os08g09840	ATGGCACGGGATATTCTAGC	AAATAGCTCTGCCCGATGAT
OsWRKY118	LOC_Os08g09900	CATGTTGGCGCCTTCTACTA	CAGTGGAATCCATCATCAGC
OsWRKY119	LOC_Os01g62510	GGATGATGGGTTGGCCTTGA	GGGTCATCGGTGGACTTCTC
OsWRKY120		AAAGACATGTGCCAGCTCCA	AGCCTTTGGGTCTTGTGAGG
OsWRKY121	LOC_Os03g53050	ACAGACGTCAAGCACTCCAG	GTGCATCGTCCACTACCATC
OsWRKY123	LOC_Os07g17230	TCCAGGAACATGCTGATTGT	CCCGCTTAGAGCCTTCATAC
OsWRKY125	LOC_Os11g45750	GGTCACTGTAGCAAGCCAAA	CATACTGGTCGATGCAATCC

Table S2. Primers used in this study

Gene	Accession no.	Sequence (5' to 3')	Experiment
OsWRKY88	Os07g40570	F: 5′- **AAAAAGCAGGCT**CGATGGCGGCCGCGCGG -3′<br>R: 5′- **AGAAAGCTGGGT**ACTAGCAAGTGATCGT -3′	Full gene cloning
Chitinase 2	Os04g41620	F: 5′-**CACC**ATGATATGGCTGTGGGTCCCA-3′<br>R: 5′-GCTGCAGCTTAGCACGTACATG-3′	Promoter cloning
Actin	XM469569	F: 5′-TCCATCTTGGCATCTCTCAG-3′<br>R: 5′-GTACCCGCATCAGGCATCTG-3′	qRT-PCR
PR10a	D38170	F: 5′-GCTACAGGCATCAGTGGTCA-3′<br>R: 5′-GACTCAAACGCCACGAGAAT-3′	qRT-PCR
Chitinase 2	Os04g41620	F: 5′- TGGGCTATCTCTCGTCCTCT -3′<br>R: 5′ TTCAGGAACGATTGCCGTGT -3′	qRT-PCR
NH1	Os01g09800	F: 5’ ATCTTGATGATGCGTTTGC -3‘<br>R: 5’ TCAGCTTGCTCCAGTATTTC -3’	qRT-PCR
AttB1<br>AttB2		F: 5′-GGGGACAAGTTTGTACAAAAAAGCAGGCT-3′<br>R: 5′-GGGGACCACTTTGTACAAGAAAGCTGGGT-3′	Vector construction
Bar		F: 5′-CCACGTCATGCCAGTTCCCG-3‘<br>R: 5′-CCATGAGCCCAGAACGACGC-3′	

The overhang sequences in these primers required for gateway vector construction are marked in bold.

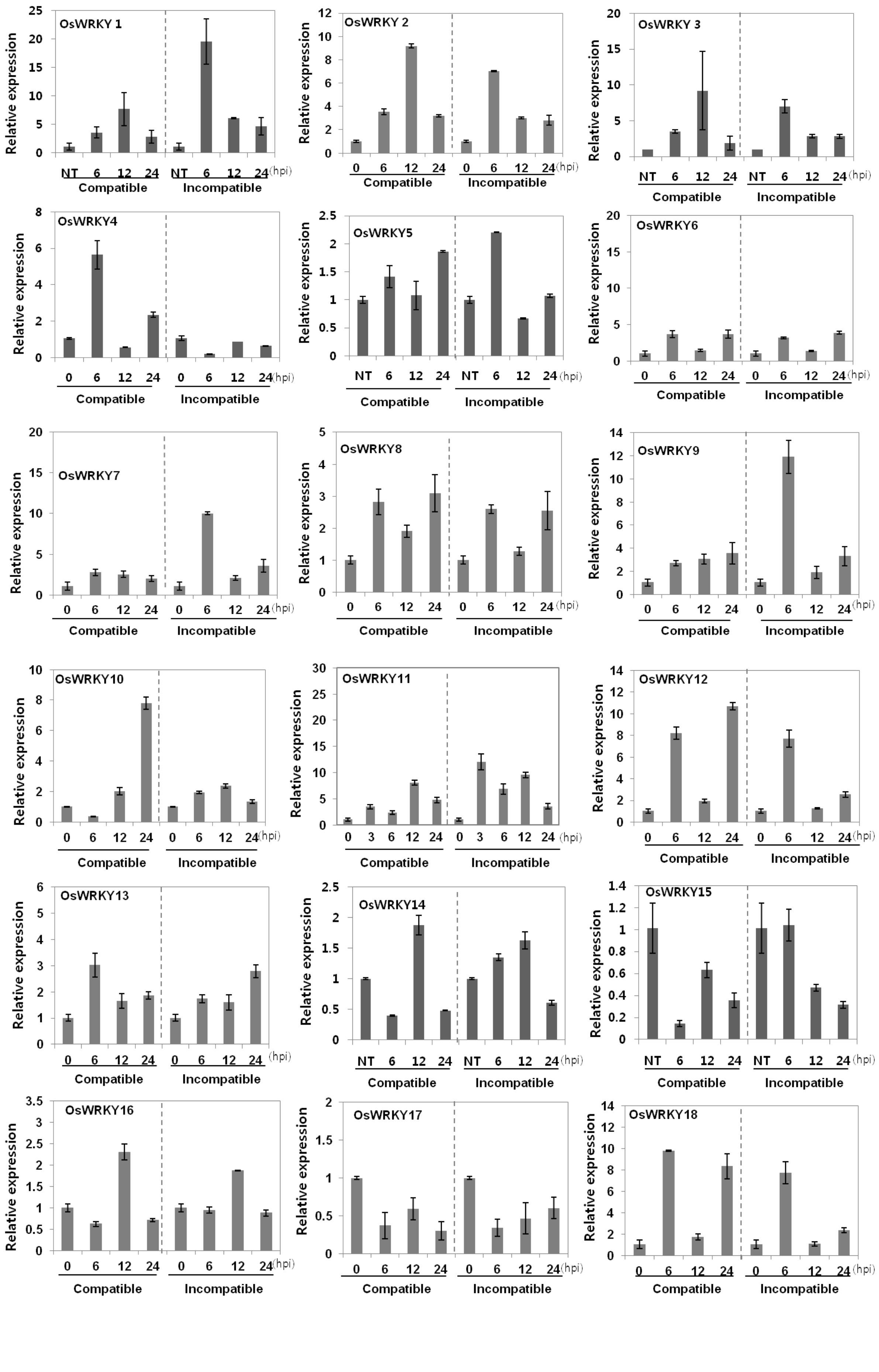

OsWRKY 1
Relative expression
NT 6 12 24 NT 6 12 24 (hpi)
Compatible
Incompatible
OsWRKY 2
0 6 12 24 0 6 12 24 (hpi)
OsWRKY 3
OsWRKY4
OsWRKY5
OsWRKY6
OsWRKY7
OsWRKY8
OsWRKY9
OsWRKY10
OsWRKY11
0 3 6 12 24 0 3 6 12 24 (hpi)
OsWRKY12
OsWRKY13
OsWRKY14
OsWRKY15
OsWRKY16
OsWRKY17
OsWRKY18

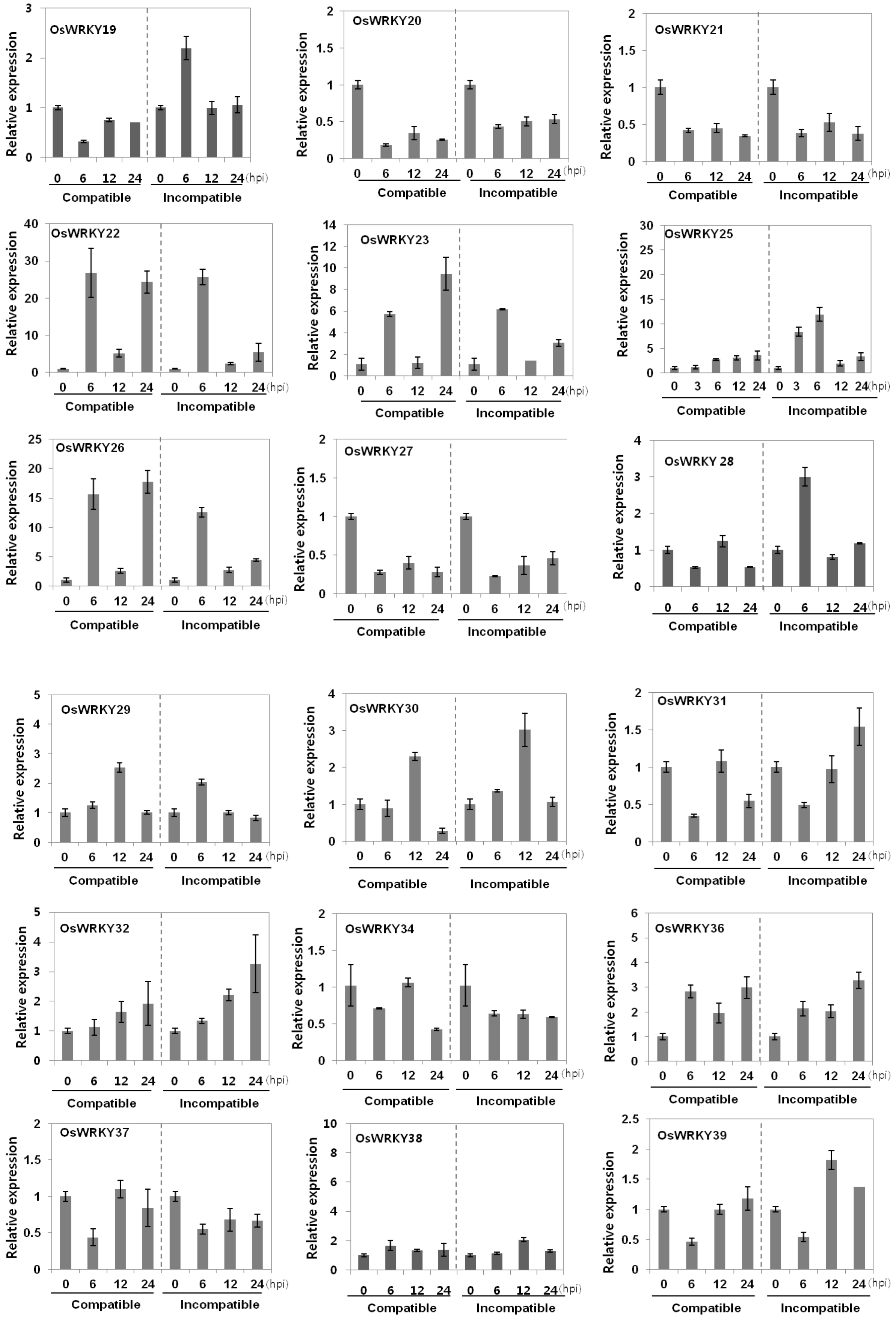

OsWRKY19
OsWRKY20
OsWRKY21
OsWRKY22
OsWRKY23
OsWRKY25
OsWRKY26
OsWRKY27
OsWRKY 28
OsWRKY29
OsWRKY30
OsWRKY31
OsWRKY32
OsWRKY34
OsWRKY36
OsWRKY37
OsWRKY38
OsWRKY39
Relative expression
0 6 12 24 0 6 12 24 (hpi)
0 3 6 12 24 0 3 6 12 24 (hpi)
Compatible
Incompatible

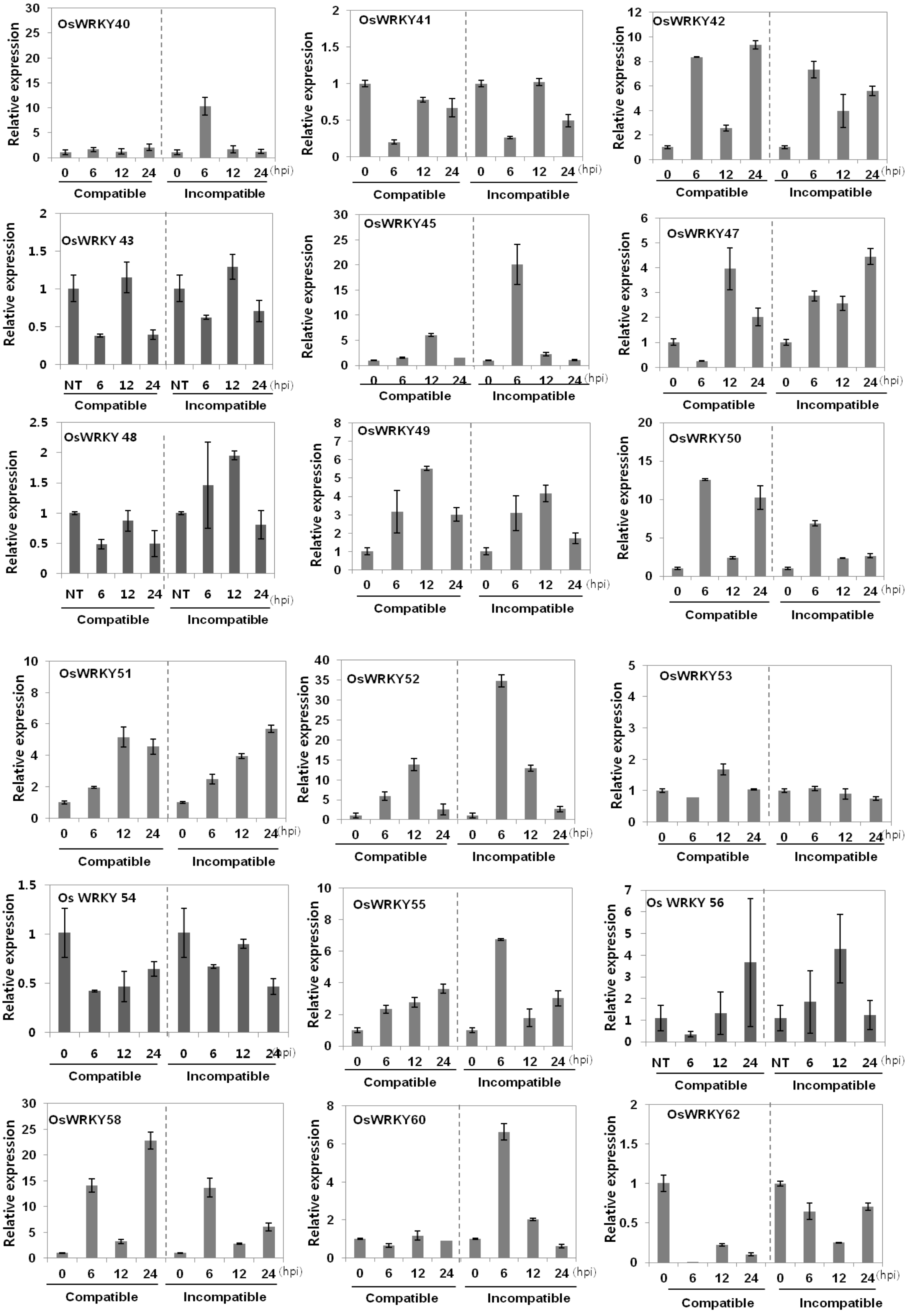

OsWRKY40
OsWRKY41
OsWRKY42
OsWRKY 43
OsWRKY45
OsWRKY47
OsWRKY 48
OsWRKY49
OsWRKY50
OsWRKY51
OsWRKY52
OsWRKY53
Os WRKY 54
OsWRKY55
Os WRKY 56
OsWRKY58
OsWRKY60
OsWRKY62
Relative expression
0 6 12 24 0 6 12 24 (hpi)
NT 6 12 24 NT 6 12 24 (hpi)
Compatible
Incompatible

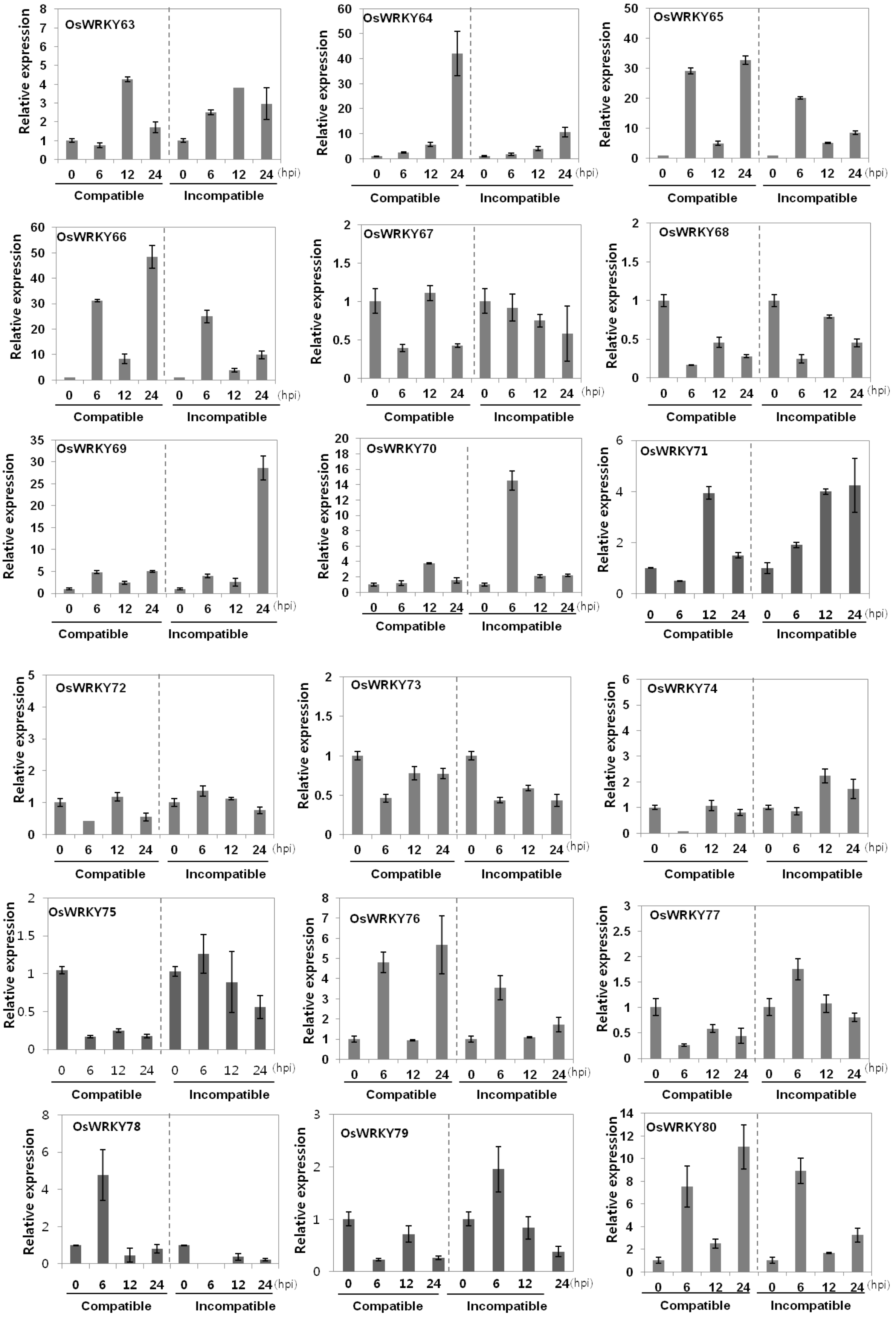

OsWRKY63
OsWRKY64
OsWRKY65
OsWRKY66
OsWRKY67
OsWRKY68
OsWRKY69
OsWRKY70
OsWRKY71
OsWRKY72
OsWRKY73
OsWRKY74
OsWRKY75
OsWRKY76
OsWRKY77
OsWRKY78
OsWRKY79
OsWRKY80
Relative expression
0 6 12 24 0 6 12 24 (hpi)
Compatible
Incompatible

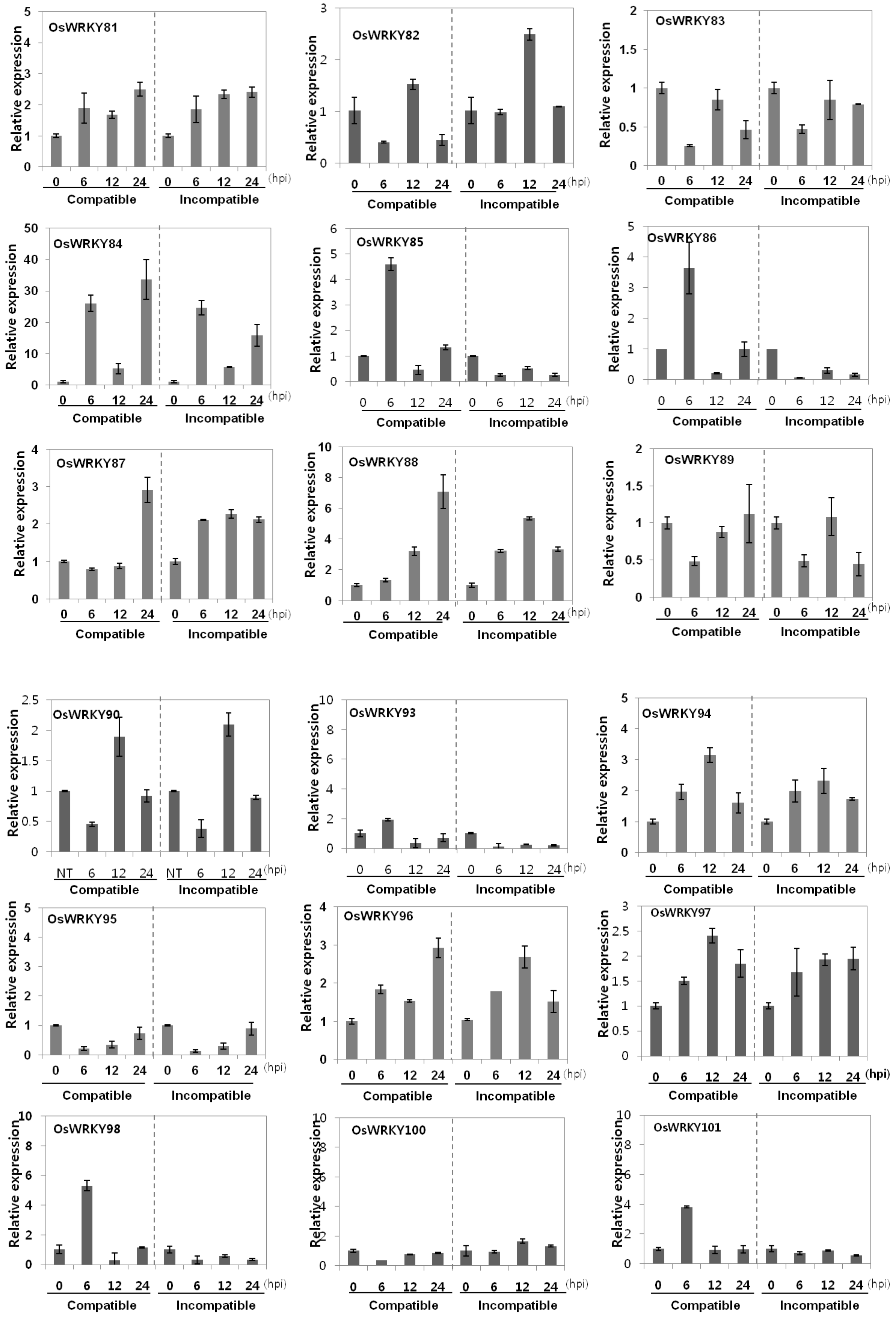
OsWRKY81
OsWRKY82
OsWRKY83
OsWRKY84
OsWRKY85
OsWRKY86
OsWRKY87
OsWRKY88
OsWRKY89
OsWRKY90
OsWRKY93
OsWRKY94
OsWRKY95
OsWRKY96
OsWRKY97
OsWRKY98
OsWRKY100
OsWRKY101
Relative expression
0 6 12 24 (hpi)
NT 6 12 24
Compatible
Incompatible

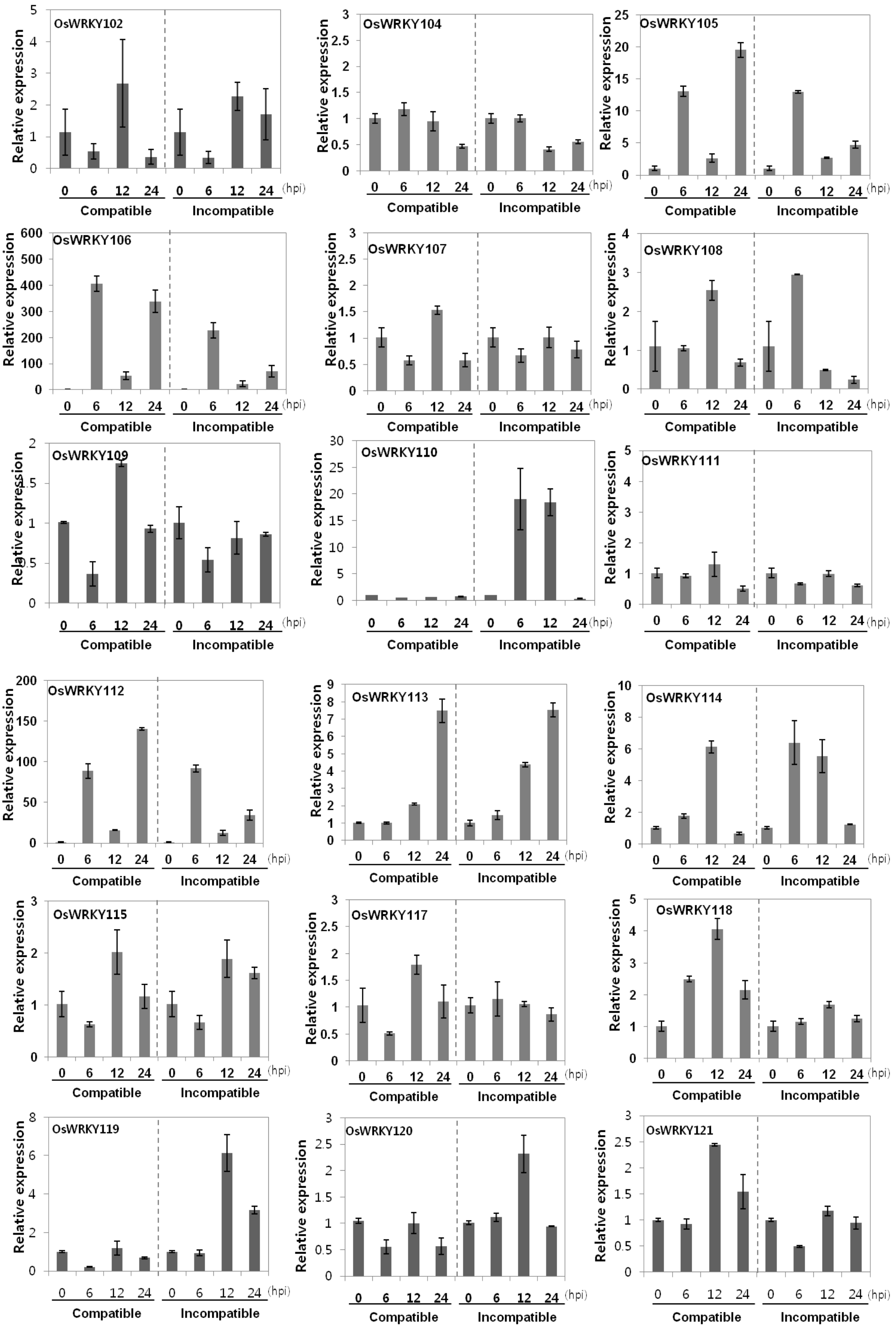
OsWRKY102
OsWRKY104
OsWRKY105
OsWRKY106
OsWRKY107
OsWRKY108
OsWRKY109
OsWRKY110
OsWRKY111
OsWRKY112
OsWRKY113
OsWRKY114
OsWRKY115
OsWRKY117
OsWRKY118
OsWRKY119
OsWRKY120
OsWRKY121
Relative expression
0 6 12 24 0 6 12 24 (hpi)
Compatible
Incompatible

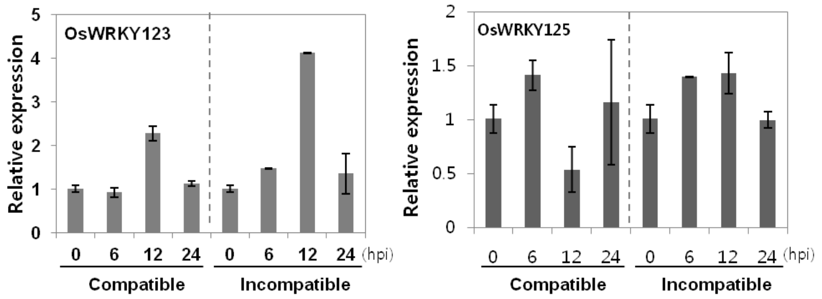

OsWRKY123
Relative expression
0
1
2
3
4
5
0
6
12
24
0
6
12
24
(hpi)
Compatible
Incompatible
OsWRKY125
Relative expression
0
0.5
1
1.5
2
0
6
12
24
0
6
12
24
(hpi)
Compatible
Incompatible

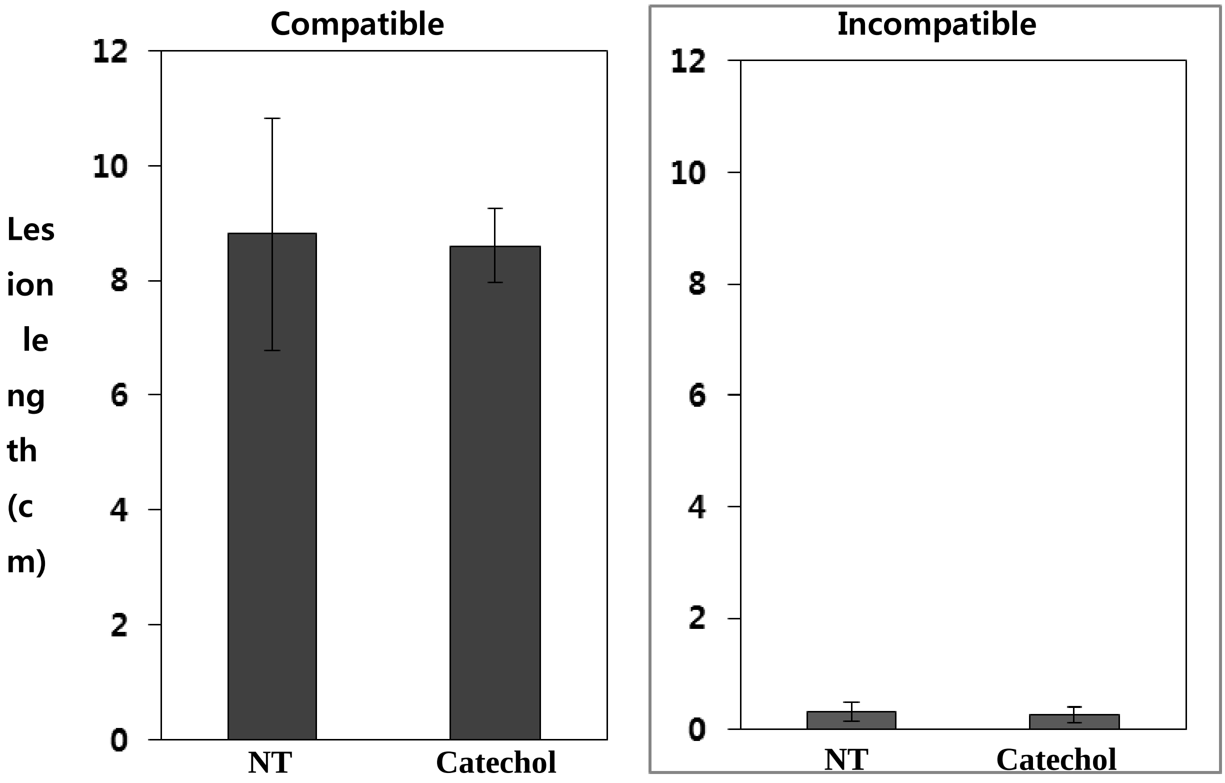

Compatible
Lesion length (cm)
0
2
4
6
8
10
12
NT
Catechol
Incompatible
0
2
4
6
8
10
12
NT
Catechol

Figure S1. The effect of catechol on compatible and incompatible interactions between *Xanthomonas oryzae* pv. *oryzae* and rice.
